# Brain signatures in children who contemplate suicide: learning from the large-scale ABCD study

**DOI:** 10.1017/S0033291721004074

**Published:** 2021-11-17

**Authors:** Andrea Wiglesworth, Conner A. Falke, Mark Fiecas, Monica Luciana, Kathryn R. Cullen, Bonnie Klimes-Dougan

**Affiliations:** 1 Department of Psychology, University of Minnesota, Minneapolis, MN, USA; 2 Division of Biostatistics, University of Minnesota, Minneapolis, MN, USA; 3 Department of Psychiatry and Behavioral Sciences, University of Minnesota, Minneapolis, MN, USA

**Keywords:** Children, default mode network, fMRI, salience network, suicide

## Abstract

**Background.:**

Suicide is the second-leading cause of death in youth. Understanding the neural correlates of suicide ideation (SI) in children is crucial to ongoing efforts to understand and prevent youth suicide. This study characterized key neural networks during rest and emotion task conditions in an epidemiologically informed sample of children who report current, past, or no SI.

**Methods.:**

Data are from the adolescent brain cognitive development study, including 8248 children (ages 9–10; mean age = 119.2 months; 49.2% female) recruited from the community. Resting-state functional connectivity (RSFC) and activation to emotional stimuli in the salience (SN) and default mode (DMN) networks were measured through fMRI. Self-reported SI and clinical profiles were gathered. We examined the replicability of our model results through repeated sub-sample reliability analyses.

**Results.:**

Children with current SI (2.0%), compared to those without any past SI, showed lower DMN RSFC (*B* = −0.267, *p* < 0.001) and lower DMN activation in response to negative as compared to neutral faces (*B* = −0.204, *p* = 0.010). These results were robust to the effects of MDD, ADHD, and medication use. Sub-sample analysis further supported the robustness of these results. We did not find support for differences in SN RSFC or in SN activation to positive or negative stimuli for children with or without SI.

**Conclusions.:**

Results from a large brain imaging study using robust statistical approaches suggest aberrant DMN functioning in children with current suicide ideation. Findings suggest potential mechanisms that may be targeted in suicide prevention efforts.

Suicide is the second-leading cause of death in young people aged 10–34 (Centers for Disease Control and Prevention (CDC), 2020). Suicide rates in youth ages 10–14 years rose 16% since 2017 (Drapeau & McIntosh, 2020). While death by suicide prior to age 10 is rare (e.g., only nine cases documented in the United States in 2018; CDC, 2018), the prevalence of suicide ideation (SI) and attempts may be higher. Although relatively high rates of suicidal thoughts and behaviors have been reported in clinical samples of young children (Dervic & Oquendo, 2019), until recently (DeVille et al., 2020), little data have been available regarding the prevalence of non-fatal suicidal thoughts and behaviors in middle childhood in the general population. Advancing our understanding of the developmental roots of suicide risk is critical for prevention efforts. It is possible that antecedents of adolescent suicide risk are evident in children with early-emerging thoughts of suicide, who have yet to engage in lethal suicide behaviors. There is a growing body of work linking brain functioning and suicidal thoughts and behaviors in adolescents and adults (e.g. Auerbach, Pagliaccio, Allison, Alqueza, & Alonso, 2021) which has stimulated research focused on suicide prevention by targeting neural mechanisms of risk (Abdelnaim et al., 2020). However, little is known about the neurobiological underpinnings of suicide risk in children. Examining patterns of brain function under multiple conditions may reveal early developmental deviations associated with child suicide risk that represent targets for intervention and prevention.

The salience (SN) and default mode (DMN) networks have been identified as particularly relevant for suicidal thoughts and behaviors in adolescents and adults (Greicius, Krasnow, Reiss, & Menon, 2003; Menon, 2015; Raichle et al., 2001) and represent a starting point for exploration of neural circuit dysfunction in children with SI. The anterior insula and dorsal anterior cingulate cortex (ACC) are prominent nodes of the SN, which integrates sensory, emotional, and cognitive information to guide behaviors particularly related to conflict monitoring and response selection (Menon, 2015). The posterior cingulate cortex, medial prefrontal cortex, and precuneus are key nodes of the DMN (Raichle, 2015), which is primarily active during rest (Greicius et al., 2003), and important for self-referential mental processing and spontaneous cognition (Andrews-Hanna, Reidler, Huang, & Buckner, 2010; Raichle, 2015).

Functional magnetic resonance imaging (fMRI) can be used to characterize the functional connectivity of different brain regions within a network and brain activation patterns in response to particular stimuli. Resting-state fMRI readily identifies the SN by the age of two, although the SN undergoes protracted developmental changes in connection strength, with increasing within-network coherence into early adulthood (Gao et al., 2013; Uddin, Supekar, Ryali, & Menon, 2011). SN dysfunction has been linked to vulnerability for psychopathology across the lifespan (Fair et al., 2013; Geng, Li, Chen, Li, & Gu, 2016; Menon, 2011, 2015; Uddin & Menon, 2009; Yuen et al., 2014). In particular, lower within-network resting-state functional connectivity (RSFC) of the SN is related to SI in adolescence (Schwartz, Ordaz, Ho, & Gotlib, 2019) and lifetime severity of SI and suicide attempts in adulthood (Cao et al., 2019; Ordaz, Goyer, Ho, Singh, & Gotlib, 2018). In addition to RSFC, fMRI paradigms that probe emotional responses can further characterize SN function in those with SI. For example, one study showed that lower activation of the anterior cingulate gyrus (a key SN node) in response to viewing angry faces was associated with past suicide attempts in adolescents (Pan et al., 2013). However, relationships between SN (either at rest or in response to emotion) and suicide risk in children remain unknown.

The DMN undergoes similar changes to the SN throughout childhood, adolescence, and into adulthood (Fair et al., 2008; Sherman et al., 2014). DMN dysfunction is implicated in most major forms of psychopathology across the lifespan (Buckner, Andrews-Hanna, & Schacter, 2008; Ho et al., 2015; Menon, 2011). Depressed adolescents with current SI demonstrated lower ventral DMN RSFC than depressed adolescents with no SI and healthy controls (Ho et al., 2021). Further, lower DMN RSFC correlated with lifetime severity of SI in adolescents with depression (Ordaz et al., 2018). In adults, lower DMN RSFC has been associated with current SI and suicide attempt history in individuals with depression (Chase et al., 2017; Jung et al., 2020). However, the developmental patterns of DMN dysfunction related to SI are unclear. Little work has examined network-level DMN activation to emotional stimuli in individuals experiencing suicidal thoughts and behaviors at any developmental stage (Auerbach et al., 2021), including children experiencing thoughts of suicide.

Examination of the brain correlates of suicidality in late childhood requires developmental considerations such as whether the particular psychological disorders that are associated with suicidality for children differ from those associated with suicidality among adolescents and adults. Sheftall et al. (2016) found that children who died by suicide were more likely than adolescents to have attention-deficit hyperactivity disorder (ADHD), which implicates ADHD as a potential risk factor for suicidality in children. Further, they found that these children were less likely than adolescents who died by suicide to have a depressive disorder (Sheftall et al., 2016). Further, ADHD, as opposed to depression, correlated with suicidality among prepubertal psychiatric inpatients and a community sample of children ages 3–7 (Goodman, Gerstadt, Pfeffer, Stroh, & Valdez, 2008; Whalen, Dixon-Gordon, Belden, Barch, & Luby, 2015). Thus, ADHD, in addition to depression, is important to consider when examining childhood suicidality.

Another developmental consideration is whether the child’s report or the parent’s report of the child’s symptomology is likely to be more accurate (e.g. see Edelbrock, Costello, Dulcan, Connover, & Kalas, 1986). With respect to internalizing psychopathology, including depression and SI, evidence suggests that the child’s report is more accurate than the parent’s (e.g. Moretti, Fine, Haley, & Marriage, 1985). Indeed, parents’ reports may be biased by their own depressive symptoms (Moretti et al., 1985). Further, self-reported depressive symptoms from children more closely align with clinicians’ diagnoses following diagnostic interviews than do parent reports of child symptoms (Cohen, So, Young, Hankin, & Lee, 2019). Findings regarding SI are similar. For child SI, parents report a lower prevalence than child self-report (Klimes-Dougan, 1998), possibly due to parents’ unawareness of their child’s SI (DeVille et al., 2020). Further, there is an inverse association between child age and parental awareness, with greater parental unawareness of SI in younger children (Jones et al., 2019). Notably, for externalizing psychopathology, such as ADHD, children tend to report fewer ADHD symptoms as compared to parents (Hennig, Schramm, & Linderkamp, 2018; Wiener et al., 2012). Therefore, reliance on parent report may be optimal. Unlike with depression, parental ADHD does not appear to add an additional source of bias to their reports of child ADHD symptoms (Faraone, Monuteaux, Biederman, Cohan, & Mick, 2003).

The Adolescent Brain Cognitive Development (ABCD) Study provides a unique opportunity to address these knowledge gaps and meet these developmental considerations, given the vast amount of data collected from a large sample of both parents and children. Vidal-Ribas et al. (2021) used the ABCD data to examine associations between lifetime and current suicidal thoughts and behaviors and brain structure, functional connectivity at rest, and activation during tasks, identifying no meaningful relationships. However, Vidal-Ribas and colleagues included parent reports in defining current SI. Given the potential bias in parent reports for internalizing symptoms, it is important to examine current SI specifically by child-report. In addition, this study tested group differences using Welch’s *t* test, which did not control for key demographic or clinical covariates that could confound results. Finally, their sample came from an earlier ABCD Study release (2.0.1) that excludes 1438 participants who were scanned using a Philips Medical Systems scanner.

The current project aims to address several important remaining knowledge gaps. First, our analytic plan accounts for various methodological and clinically relevant effects. Second, we examine both past and current SI, to explore whether brain-behavior relationships better reflect transient states or persistent traits. Third, we expand our sample by using the corrected Philips data. Specifically, we will examine the relation between past and current SI based on the child’s self-report and SN and DMN within-network RSFC and activation while viewing emotional *v*. neutral stimuli. We predict that children with both past and current SI will demonstrate lower within-network RSFC of the SN and DMN as compared to peers, but that this effect will be stronger in children with current SI. We also predict that the SN and DMN will demonstrate different activation patterns for children with and without both lifetime and current SI when viewing emotional stimuli. However, because the limited research in this area has focused primarily on activations in response to angry faces (Auerbach et al., 2021), *v*. other negatively valenced emotions, we do not predict the direction of this group difference.

## Method

### Procedure

The ABCD Study® is a multisite study which recruited an epidemiologically informed sample of 9- to 10-year-old children at baseline (Garavan et al., 2018). The study aims are to inform our understanding of the environmental, genetic, neurobiological, and behavioral factors related to youth mental and physical health (Jernigan & Brown, 2018). We utilized baseline data from ABCD’s version 3.0.1 data release (October 2020).

### Participants

The ABCD Study® sample includes 11 887 children and their parents/guardians. Participants were excluded from current analyses if they had missing relevant demographic, brain imaging, or clinical data (see consort diagram, online Supplementary Fig. S1). The final sample included 8248 9- to 10-year-old children (mean age =119.21 months/ 9.93 years old). The sample was 49.21% assigned female sex at birth; 2247 individuals had a sibling in the study (997 non-twin siblings, and 1250 twins or triplets).

### Measures

#### KSADS

Children and parents completed the computerized Kiddie Schedule for Affective Disorders and Schizophrenia for DSM-5 (KSADS-5) to assess SI and other relevant diagnoses. Children were coded as having SI if they endorsed suicidal ideation in any of the following five domains: passive, active but non-specific, specific method, active with intent, and active with a plan (for full wording of KSADS SI questions, and participant breakdown of SI endorsement, see online Supplementary Table S1). Current SI was coded if children reported having SI in the past 2 weeks. Past SI was coded if they endorsed previous thoughts of suicide in their lifetimes. Children were coded as having no, past, or current ADHD based on the *parent report* of ADHD symptoms and no, past, or current MDD if they met diagnostic criteria through the *child report*.

#### fMRI data acquisition

**Fig. 1.**
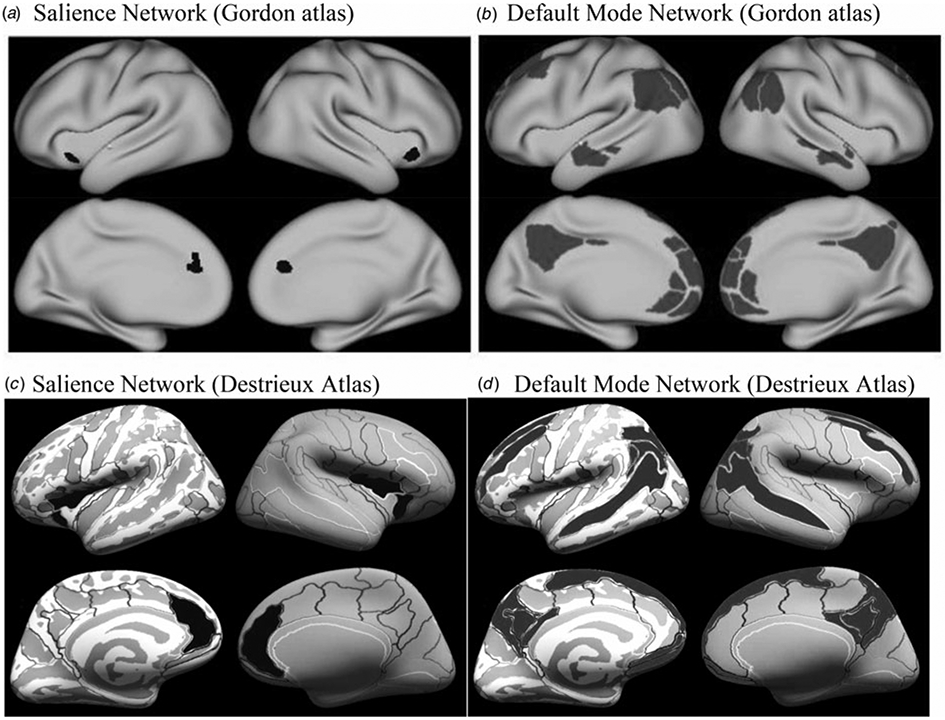
Salience and Default mode networks parcellations as defined by the Gordon and Destrieux atlases. (*a*) Salience Network (Gordon atlas); (*b*) Default Mode Network (Gordon atlas); (*c*) Salience Network (Destrieux Atlas); (*d*) Default Mode Network (Destrieux Atlas).

Detailed descriptions of ABCD imaging acquisition, processing, quality assessment, and analysis have been described elsewhere (Casey et al., 2018; Hagler et al., 2019). Imaging data were acquired across 21 study sites and harmonized across three 3T scanner platforms (Siemens Prisma, Philips, General Electric 750). Resting-state scans comprised 20 minutes of scanning collected across four runs. Participants were instructed to keep their eyes open and view a cross-hair. The DMN and SN were defined by the Gordon atlas (Fig. 1a and b; Gordon et al., 2016) in the ABCD Study data. We used pretabulated data provided by the ABCD Study, where within-network resting-state values were calculated by taking the average of all pairwise correlations of all combinations of regions of interest (ROI) (DMN *n* = 41 regions, SN *n* = 4 regions) for each network. Resting-state correlation values were Fisher’s Z transformed (Hagler et al., 2019).

Task-fMRI data were used to examine DMN and SN activations during the processing of positive (happy) or negative (fearful) emotional face stimuli in contrast to activation during the processing of neutral stimuli through the Emotional N-Back task (Cohen, Conley, Dellarco, & Casey, 2016; see Hagler et al., 2019). We utilized the pretabulated data released by the ABCD Study, which included activation values in ROIs derived from positive-neutral and negative-neutral contrasts of the Emotional N-back, averaged over all trials and runs. The Destrieux atlas was used to parcellate the brain regions for the task-fMRI data (Destrieux, Fischl, Dale, & Halgren, 2010). To define the DMN and SN for the task-fMRI, the Gordon and Destrieux atlases were compared for overlapping regions through independent visual inspection performed by two authors (Fig. 1c and d; online Supplementary Table S2). Functional activation values in regions identified within the Destrieux Atlas as being part of the DMN (*n* = 12 regions) and SN (*n* = 3 regions) were averaged to create a single network value. We included only data from individuals who had a Freesurfer quality control score of ‘acceptable’ across resting state and task scans (Hagler et al., 2019).

### Data analytic plan

Analyses were conducted in R version 4.0.2. A series of 2-tailed chi-square analyses and ANOVA F-tests were conducted to examine differences in demographic factors, MDD, and ADHD between children with current SI, children with prior SI histories, and children without any history of SI. We ran linear mixed-effect regression models (LMER) using R packages lme4 and lmerTest and visually assessed the normality and linearity of our results per LMER assumptions. We used LMER to examine relations between past and current SI, as compared to no SI, and SN and DMN RSFC, and associations between indices of SI and SN and DMN activations during emotional (positive; negative) *v*. neutral EN-back conditions. All models included fixed effects covariates to control for differences in connectivity and activation by demographic factors (age, sex, race, and family income band) and fMRI scanner platform (Hagler et al., 2019 for recommendation) as well as random effects for research site (22 groups) and family nested within site (7116 groups) (e.g., lme 4 notation of (1∣ siteID/familyID)). Notably, due to low numbers of current SI participants in many subgroups we do not include the interaction of SI and our fixed effect covariates, and therefore can not calculate the effects of SI in specific subgroups. All LMER were run an additional time controlling for MDD and ADHD, both past and present, as well as prescriptions for antidepressant and stimulant medications (see supplemental method). Cohen’s *f* ^2^ (Selya, Rose, Dierker, Hedeker, & Mermelstein, 2012) effect size estimates for LMER are reported in the data tables. When performing ROI-level post-hoc tests, we corrected the *p*-values for multiple comparisons using the Hochberg procedure and the R package stats (Hochberg, 1988).

Given concerns that our relatively small subsample of youth who endorse current SI may produce irreplicable findings, we wanted to verify that observed effects remained when looking at different samples. To achieve this, we conducted a repeated sub-sample analysis for each network-wide LMER (examining the relationship between the network measure and SI) both before and after controlling for MDD and ADHD. Each sub-sample consisted of half the total participants and was created by randomly selecting families in a stratified manner to ensure the halves were balanced with regard to the number of participants from each ABCD Study site, numbers with current SI, and numbers with past SI. We then conducted the network-wide LMERs in each half and saved the standardized beta coefficient for current SI. We repeated the sampling and analysis 100 times and reported the 2.5 and 97.5 percentiles of the beta coefficients from the LMER analyses across the 100 iterations.

## Results

### Participants

Descriptive statistics and group differences in demographic and clinical variables are presented in Table 1. One-hundred sixty-four children (2.0% of the sample) endorsed current SI and 496 children (6.0% of the sample) endorsed past SI. Of those with current SI, 13 (7.9%) met criteria for current MDD; 27 (16.5%) met criteria for current ADHD; three (1.8%) met criteria for both. Reports of MDD and ADHD, both current and past, were significantly different across SI groups (*p*’s < 0.001).

### Resting-state functional connectivity

LMER revealed that neither past (*B* = 0.011, *p* = 0.805) nor current (*B* = −0.146, *p* = 0.064) SI were significantly associated with lower within-network SN RSFC when compared to no SI youth (online Supplementary Table S3). These results remained not significant in a follow-up analysis controlling for MDD, ADHD, antidepressant medication, and stimulant medication. Current SI was significantly associated with lower within-network DMN RSFC (*B* = −0.267, *p* < 0.001) as compared to no SI, and remained significant (*B* = −0.222, *p* = 0.004) when controlling for both current or past MDD and ADHD as well as an antidepressant and stimulant medication (Table 2). However, there were no significant differences between no and past SI in DMN RSFC in either model. Results from sub-sample analyses indicate that, even when looking across 100 random samples, observed effects are the same in size and direction (Table 3).

We conducted post-hoc regressions to probe both the SN and DMN for pairwise resting-state connections between individual ROIs for a finer-grained understanding of these networks and potential relationships to current SI. No individual pairwise connections were significantly associated with current SI, as compared to no SI, within the SN after controlling for the six tests (online Supplementary Fig. S2). With respect to the DMN, no pairwise connections were significantly related to current SI, as compared to no SI, after controlling for the 820 comparisons. However, pairwise connections between the dorsal medial prefrontal cortex and the middle temporal gyrus of the DMN demonstrated the largest associations with current SI as compared to no SI (*B* = −0.458, *P*_hoch_ = 0.094; online Supplementary Fig. S3).

### Task-fMRI activation

LMER revealed that SN activation during positive *v*. neutral (*B*_past_ = −0.048, *p* = 0.303; *B*_present_ = 0.064, *p* = 0.417) or negative *v*. neutral (*B*_past_ = −0.084, *p* = 0.071; *B*_present_ =−0.032, *p* = 0.688) contrasts were not significantly related to past or current SI, as compared to no SI (online Supplementary Tables S4 and S5). Current SI was associated with lower DMN activation during negative-neutral contrasts of the E-NBack (*B* = −0.204, *p* = 0.010; Table 4), as compared to no SI. After winsorizing the most extreme 10% activation values, current SI remained significantly associated with lower DMN activation during negative-neutral contrasts as compared to no SI (*B* = −0.098, *p* = 0.044), but this difference became nonsignificant when controlling for clinical variables (*B* = −0.088, *p* = 0.066). Past and current SI were not significantly related to DMN activation during positive-neutral contrasts (*B*_past_ = −0.066, *p* = 0.152; *B*_present_ = −0.115, *p* = 0.147) when compared to no SI (online Supplementary Table S6). All findings generally showed acceptable replication in the sub-sampling analysis (Table 3).

We conducted additional post-hoc tests to probe the SN and DMN for activation in ROIs that may, on their own, be related to current SI. We found no significant differences between no SI and current SI in the SN when controlling for the six tests of different region/contrast pairs (online Supplementary Table S7). However, in the DMN, activation during the negative-neutral contrasts in the superior temporal sulcus (*B*_present_ = −0.271, *p*_hoch-_ = 0.014), posterior dorsal cingulate gyrus (*B*_present_ = −0.264, *p*_hoch_ = 0.019), and subparietal sulcus (*B*_present_ =−0.254, *p*_hoch_ = 0.029) were all significantly negatively associated with present SI compared to no SI after controlling for the testing of 24 region/contrast pairs (online Supplementary Table S8).

## Discussion

The ABCD Study® provides an important opportunity to understand typical and atypical neurodevelopmental processes. Capitalizing on the study’s considerable size and scope allowed us to conduct the largest characterization of SN and DMN functional connectivity and activation in children who have recently experienced SI to date. Additional strengths of this investigation included the application of a multi-modal approach for examining SN and DMN during both rest and task, the inclusion of clinical covariates, and the focus on child self-reports of past and current SI. The ABCD Study shows evidence that SI is present even in children as young as 9-years-old (Blashill & Calzo, 2019; DeVille et al., 2020), thereby underscoring the importance of identifying neural correlates of SI during childhood, prior to the onset of lethal suicide attempts. The results of this study suggest that patterns of atypical DMN functioning at rest and during a task, which are associated with suicidality in adolescence and adulthood, are also evident in middle-to-late childhood in those with current, but not past, SI.

Indeed, in our study, children who endorsed having thoughts of suicide within the past 2 weeks demonstrated significantly lower RSFC of the DMN as compared to their non-ideating peers, and this pattern was still significant above and beyond the effects of ADHD, MDD, and medications. These findings align with past research examining DMN RSFC in individuals with SI, which has focused primarily on adolescents and adults with depression diagnoses (Chase et al., 2017; Jung et al., 2020; Ordaz et al., 2018). Given that the DMN is implicated in self-referential processing and understanding of one’s self in the world (Raichle, 2015), low DMN RSFC might reflect impairment in some of these essential meaning-making processes in people with depression and SI, including children. Interestingly, our results largely mirror those found in a previous study of the ABCD population (Vidal-Ribas et al., 2021). However, the methodological differences between their project and ours allowed us to identify an important, notable, and replicable dysfunction in the DMN in children with current SI.

As this finding derives from cross-sectional data, we cannot definitively say whether DMN resting-state abnormalities associated with current SI represent a delay in the typical developmental trajectory of DMN RSFC, in which within-network connectivity has been observed to increase across childhood and adolescence (Fair et al., 2008; Gao et al., 2013; Sherman et al., 2014; Uddin et al., 2011), or if they represent a more transient, state-like vulnerability that change as children move in and out of states of suicidality. However, the patterns of our findings lend themselves to the hypothesis of a state-like vulnerability, given that current, but not past, SI was associated with lower DMN RSFC. Longitudinal research, preferably with a more sensitive time frame than that available from the ABCD Study, would be necessary to establish evidence for this hypothesis.

With DMN RSFC being implicated in suicidal thoughts and behaviors in childhood, adolescence, and adulthood, RSFC of these networks may be a reasonable target for prevention or intervention efforts. For example, cognitive training aimed at increasing DMN RSFC beginning in childhood may be an avenue for future study, as suggested by research in older adult populations showing that cognitive training can modulate and strengthen DMN RSFC (Cao et al., 2016). Another technique, neuromodulation, may be promising in targeting DMN RSFC in children, as it has been shown to reduce suicidality and modulate DMN RSFC in older samples (Abdelnaim et al., 2020; Keeser et al., 2011; Xiao et al., 2020).

We also examined SN and DMN activation during the EN-back, the only task in the ABCD study that assesses MRI-based brain functioning when viewing emotional stimuli, which also includes working memory. Consistent with our predictions, children with current SI demonstrated lower DMN activation while viewing negative *v*. neutral faces as compared to their peers with no ideation history. This network finding appeared to be driven by decreased activation in the regions near the superior temporal sulcus, posterior dorsal cingulate gyrus, and subparietal sulcus. Given that the DMN demonstrates patterns of hypoactivation during the processing of negative, as opposed to neutral stimuli in children with current SI, this type of emotional processing may represent an additional target for intervention. Parental support during the processing of negative emotional events may be helpful in scaffolding responses to these stimuli. Indeed, research has indicated that parental support may be especially helpful for children in regulating their physiological reactions to stressful experiences (Hostinar, Johnson, & Gunnar, 2015). The same might be true for areas of emotional processing.

We failed to find a significant relationship between SN RSFC or task activation and current or past SI, as compared to non-ideating peers. However, SN RSFC, while nonsignificant, did show a relationship with current SI in the expected direction, and these findings would have emerged as significant under a critical alpha level of *p* < 0.1. These findings may represent the very beginnings of dysfunction, which may become more pronounced in time. In tandem with past research, they may imply that associations between RSFC of the SN and SI are not detectable until later in development, i.e., adolescence or adulthood (Cao et al., 2019; Ordaz et al., 2018; Schwartz et al., 2019). Future iterations of this work within the ABCD Study will be able to examine this same group of children longitudinally to identify the developmental period in which this established relationship between SN RSFC and SI emerges.

Contrary to predictions, we found no significant relationships between SI and SN activation during an emotionally salient task, potentially suggesting that there are no links between brain activations to emotional stimuli in the SN and SI for this age group. However, these results may be reflective of this particular task, rather than emotional processing in general, as research that has identified SI group differences in task activation more commonly involve activation of the salience and threat systems (e.g. Jollant, Lawrence, Olié, Guillaume, & Courtet, 2011; see also Olié et al., 2017).

Importantly, demographic differences were noted in child self-reports of SI. Both Native American and Latinx/Hispanic youth were more likely than youth from other racial or ethnic groups to report SI (Table 1), though there was not a relationship between racial identity and DMN or SN function for these youth. Individuals in our study who endorsed SI had significantly lower family income than those who did not endorse SI, and lower family income was associated with lower SN and DMN RSFC. While suicide affects individuals from all socioeconomic backgrounds, research has shown that people from low socioeconomic neighborhoods or backgrounds are more likely to experience SI and suicide attempts, including fatal attempts (Burrows & Laflamme, 2010; Dupéré, Leventhal, & Lacourse, 2009; Rehkopf & Buka, 2006). Future research should examine how socioeconomic inequalities are biologically embedded to lead to alterations in key neural network functioning and increased suicide risk prior to adolescence.

### Limitations

One notable feature of this study is the epidemiologically informed approach to systematically sampling brain functioning in a large group of 9- to 10-year-old children. The current project highlights the importance of large-scale neuroimaging studies, particularly when identifying neural correlates of suicidal thoughts and behaviors in this developmental period. Nevertheless, concerns with respect to power should be highlighted given that SI in children is rare and our sample of youth with current SI make up a small proportion of the total ABCD Study sample. While it is notable that our sample of youth with current SI is approximately four times larger than those included in previous studies identifying neural biomarkers of suicidal thoughts and behaviors in youth (see Auerbach et al., 2021 for review), these findings were based on 164 youth who reported current SI and results should be interpreted accordingly. One particular constraint of small sample sizes is the inability to detect SI effect sizes in subgroups of interest, e.g., racial subgroups. To address the concern that our relatively small current SI sample may inappropriately yield results that are not replicable, we bolstered our findings by completing 100 sub-sample replications, which generally showed good replication in the size and direction of regression coefficients. To provide further confidence that our results were not driven by outliers, we confirmed that our models generally remained significant when winsorizing the most extreme 10% of connectivity and activation values. The developmental stability of these findings awaits further evidence and can be addressed when longitudinal data from the ABCD Study® become available.

Suicide assessment in childhood requires making a variety of decisions, which may act as limitations. This analysis relied on data from the KSADS, which queries a variety of passive and active suicidal thoughts. While there is evidence that children by age 9 may have an understanding of life, death, and suicide (Kenyon, 2001), we may expect passive SI (e.g., “I would be better off dead”) and active ideation (e.g., “I want to kill myself”) to be qualitatively different. Notably, our results remained the same when looking at youth with any SI, only active SI (see online Supplementary Tables S9-S13), and SI in the absence of suicidal behavior (online Supplementary Tables S14-S18). While the child’s self-report of suicidality appeared robust, the same might not be true for the parent report. Indeed, in our sample, combined parent and child report produced results similar to those defined by the child only, though with smaller effects (online Supplementary Tables S19-S23). However, when looking at parent-reported SI alone, there were no significant relationships identified between brain function and child suicidality (online Supplementary Tables S24-S28). Such results indicate that RSFC and task-activation in the DMN are more linked to child-reported SI than to parent-reported SI. More research that incorporates self-reported SI along with clinician diagnosis is needed to link this finding to past research, which suggests that parents may be largely unaware of child suicidality (including the severity or recency; DeVille et al., 2020; Jones et al., 2019; Klimes-Dougan, 1998). Given this evidence, we believe that our choice to rely on child-reported suicidality was a strength of this study and that future research would benefit most from trusting child-reported suicidality alone or engaging in a consensus process that combines parent and child report.

As this study utilized pretabulated fMRI data from ABCD’s NDA 3.0 release, our characterization of the DMN and the SN could not be identical for resting-state fMRI (which used the Gordon atlas) and the task fMRI results (which used the Destrieux atlas). Considering both resting-state and task analyses provides insight into how the SN and DMN operate under different conditions, however, these findings must be interpreted with the understanding that the boundaries of the networks differ slightly as defined by these different parcellations. Additionally, while the EN-back provided a useful starting place to examine emotion processing, alternate fMRI tasks might have been more suitable for probing the SN and DMN. Finally, the cross-sectional design of these analyses precludes the ability to determine the directionality of the reported associations. Longitudinal assessment as provided by future examinations of the ABCD Study cohort will be required to fully characterize developmental trajectories and the long-range clinical impacts of our DMN findings.

## Conclusion

Suicide is the second leading cause of death for young people aged 10–34 (CDC, 2020). We report that in an epidemiologically informed sample of 9- to 10-year-old children, 8.0% have already contemplated suicide, with 2.0% having these thoughts within the 2 weeks prior to the study visit. In this developmental cohort, prior to the typical onset of fatal suicide attempts, neural signatures of the default mode network during multiple conditions are associated with current, but not past, SI. These data fill an important gap on neural correlates of suicide risk in this young age range. The identification of these mechanisms is critical if we are to capitalize on the opportunity for prevention and intervention of suicidal thoughts and behaviors.

## Supplementary Material

Supplementary Material

## Figures and Tables

**Table 1.** Sample descriptors based on group status and results from two-tailed chi-squared and Independent samples *t* test analyses to determine group differences

	No SI (*n* = 7588)	Past SI (*n* = 496)	Current SI (*n* = 164)	*p* value
Age in months, M (s.d.)	119.21 (*7.52*)	119.65 (*7.58*)	117.63 (*6.89*)	0.01
Total income, M (s.d.)^a^	7.38 (*2.34*)	7.35 (*2.22*)	6.91 (*2.53*)	0.04
Sex, *N* (%)				0.03
Male	3825 (*50.41*)	267 (*53.83*)	97 (*59.14*)	
Female	3763 (*49.59*)	229 (*46.17*)	67 (*40.85*)	
Race/Ethnicity, *N* (%)^b^				
White	5979 (*78.80*)	384 (*77.42*)	121 (*73.78*)	0.24
Black/African American	1333 (*17.57*)	100 (*20.16*)	32 (*19.51*)	0.29
Native American	233 (*3.07*)	24 (*4.84*)	9 (*5.49*)	0.02
Asian	522 (*6.88*)	38 (*7.66*)	13 (*7.93*)	0.71
Other race	451 (*5.94*)	24 (*4.84*)	11 (*6.71*)	0.54
Hispanic/Latinx	1475 (*19.44*)	74 (*14.92*)	41 (*25.00*)	0.01
Antidepressant Medication	109 (*1.44*)	36 (*7.26*)	6 (*3.66*)	<0.001
Stimulant medication	528 (*6.96*)	61 (*12.30*)	18 (*10.98*)	<0.001
MDD past, *N* (%)	87 (*1.15*)	34 (*6.85*)	10 (*6.10*)	< 0.001
MDD current, *N* (%)	31 (*0.41*)	7 (*1.41*)	13 (*7.93*)	< 0.001
ADHD past, *N* (%)	639 (*8.42*)	75 (*15.12*)	24 (*14.63*)	< 0.001
ADHD current, *N* (%)	735 (*9.69*)	75 (*15.12*)	27 (*16.46*)	< 0.001
Sibling, *N* (%)^c^	1943 (*25.61*)	26 (*5.24*)	6 (*3.66*)	< 0.001
Twin, *N* (%)^d^	1087 (*15.22*)	8 (*1.61*)	5 (*3.05*)	< 0.001

SI, suicide ideation; MDD, major depressive disorder; ADHD, attention-deficit hyperactivity disorder.

a Total income bands: 1 = Less than $5000; 2 = $5000–$ 11 999; 3 = $ 12 000–$ 15 999; 4 = $ 16 000–$ 24 999; 5 = $ 25 000–$ 34 999; 6 = $ 35 000–$ 49 999; 7 = $ 50 000–$ 74 999; 8 = $ 75 000–$ 99 999; 9 = $ 100 000–$ 199 999; 10 = $ 200 000 and greater.

b We allowed for participant overlap in the coding of racial or ethnic identity, where multiracial/multiethnic participants are represented in all relevant categories.

c Number of participants with a twin or non-twin sibling within the same SI group.

d Number of participants with a twin in the same SI group.

**Table 2.** Linear Mixed-Effects Models of the association between Default Mode network within-network RSFC and SI without and with controlling for the effects of current major depressive disorder, attention-deficit hyperactivity disorder, and medications

	Default mode network resting-state functional connectivity			
	Model 1		Model 2	
Main effects	*B*	(s.e. b)	*B*	(s.e. b)
Age (in months)^a^	0.05***	(0.01)	0.05***	(0.01)
Sex (REF = Female)	−0.25***	(0.02)	−0.25***	(0.02)
Race				
White	0.04	(0.04)	0.04	(0.04)
Black	−0.20***	(0.04)	−0.20***	(0.04)
Asian	−0.01	(0.04)	−0.02	(0.04)
Native American	0.06	(0.06)	0.06	(0.06)
Hispanic/Latinx	−0.02	(0.03)	−0.02	(0.03)
Other Race	−0.01	(0.05)	−0.01	(0.05)
Family income	0.02	(0.01)	0.01	(0.01)
Antidepressant medication	–	–	−0.08	(0.08)
Stimulant medication	–	–	0.07	(0.05)
MDD (REF = No MDD history)				
Past	–	–	−0.06	(0.09)
Current	–	–	−0.46***	(0.14)
ADHD (REF = No ADHD history)				
Past	–	–	−0.05	(0.04)
Current	–	–	−0.10**	(0.04)
Suicide ideation (REF = No SI history)				
Past	0.04	(0.04)	0.06	(0.05)
Current	−0.27***	(0.08)	−0.22**	(0.08)

SI, suicide ideation; DMN, default mode network; MDD, major depressive disorder; ADHD, attention-deficit hyperactivity disorder; REF, Reference group for categorical variables. Primary Covariates *F*^2^: 0.001538019, Secondary Covariates *F*^2^: 0.001467613.

a Pubertal status was also tested in addition to and in place of age. Results remained unchanged.

Note:

* *p* < 0.05

** *p* < 0.01

*** *p* < 0.001.

**Table 3.** Sub-sampling analyses of the standardized beta values representing the effect size of the relationship between current suicide ideation and the primary resting-state functional connectivity and task activation analyses

		Sub-sample percentiles (*B*)	
Scan	Covariates	2.5 percentile	97.5 percentile
Salience network			
Resting state	Model 1	−0.268	−0.015
Resting state	Model 2	−0.254	−0.013
Task: PVN	Model 1	−0.235	0.243
Task: PVN	Model 2	−0.254	0.244
Task: NVN	Model 1	−0.109	0.161
Task: NVN	Model 2	−0.126	0.158
Default mode network			
Resting state	Model 1	−0.387	−0.131
Resting state	Model 2	−0.344	−0.084
Task: PVN	Model 1	−0.262	0.012
Task: PVN	Model 2	−0.282	0.006
Task: NVN	Model 1	−0.444	−0.009
Task: NVN	Model 2	−0.474	−0.053

PVN, Positive *v*. neutral task contrasts; NVN, Negative *v*. neutral task contrasts.

*Note:* Covariates included in model 1 analyses are age, sex, race, family income band, and fMRI scanner platform. Model 2 analyses include all covariates from model 1 as well as antidepressant medication, stimulant medication, major depressive disorder, and attention-deficit hyperactivity disorder.

The 2.5 and 97.5 percentiles represent the range for the standardized beta value in the middle 95% of the 100 independent sub-sample iterations.

**Table 4.** Linear mixed-effects models examining the association between current SI and DMN activation during negative-*v*.-neutral contrasts of the Emotion N-back task without and with controlling for the effects of current MDD and ADHD

	DMN Negative V neutral task activation			
	Model 1		Model 2	
Main effects	*B*	(s.e. b)	*B*	(s.e. b)
Age (in months)^a^	0.00	(0.01)	0.00	(0.01)
Sex (REF = Female)	0.00	(0.02)	−0.01	(0.02)
Race				
White	0.05	(0.04)	0.04	(0.04)
Black	0.07	(0.04)	0.07	(0.04)
Asian	0.01	(0.05)	0.01	(0.05)
Native American	−0.08	(0.06)	−0.08	(0.06)
Hispanic/Latinx	−0.05	(0.03)	−0.05	(0.03)
Other Race	0.14*	(0.06)	0.14*	(0.06)
Family Income	−0.01	(0.01)	−0.01	(0.01)
Antidepressant Medication	–	–	0.06	(0.08)
Stimulant Medication	–	–	0.04	(0.05)
MDD (REF = No MDD History)				
Past	–	–	0.05	(0.09)
Current	–	–	0.09	(0.14)
ADHD (REF = No ADHD History)				
Past	–	–	0.06	(0.04)
Current	–	–	0.05	(0.04)
Suicide ideation (REF = No SI History)				
Past	−0.03	(0.05)	−0.05	(0.05)
Current	−0.20**	(0.08)	−0.22**	(0.08)

SI, suicide ideation; SN, salience network; MDD, major depressive disorder; ADHD, attention-deficit hyperactivity disorder; REF, Reference group for categorical variables. Primary covariates *F*^2^: 0.000763398, Secondary covariates *F*^2^: 0.001104355.

a Pubertal status was also tested in addition to and in place of age. Results remained unchanged.

Note:

* *p* < 0.05

** *p* < 0.01

*** *p* < 0.001.
