# Supplementary material for: Brain signatures in children who contemplate suicide: learning from the large-scale ABCD study
Source: Psychol Med. Author manuscript; Available in PMC 2023 Jun 14. (PMC10106301; doi:10.1017/S0033291721004074)
Supplement: Supplementary Material [file NIHMS1773959-supplement-Supplementary_Material.docx]

Brain signatures in children who contemplate suicide: learning from the large-scale ABCD study

# Supplementary Materials

[**Supplemental Method.**](#_30j0zll) **5**

[**Table S1. Kiddie Schedule of Affective Disorders and Schizophrenia (KSADS) suicide ideation measures, and distribution of the study sample, across the following domains: passive, active but non-specific, specific method, active with intent, and active with a plan.**](#_1fob9te) **6**

[**Table S2. Destrieux Atlas SN and DMN network regions**](#_2et92p0) **7**

[**Table S3. Linear Mixed-Effects Models of the association between salience network within-network RSFC and SI without and with controlling for the effects of current Major Depressive Disorder and Attention-Deficit Hyperactivity Disorder.**](#_tyjcwt) **8**

[**Table S4. Linear mixed-effects models examining the association between current SI and SN activation during positive-versus-neutral contrasts of the Emotion N-back task without and with controlling for the effects of current MDD and ADHD.**](#_3dy6vkm) **9**

[**Table S5. Linear mixed-effects models examining the association between current SI and SN activation during negative-versus-neutral contrasts of the Emotion N-back task without and with controlling for the effects of current MDD and ADHD.**](#_1t3h5sf) **10**

[**Table S6. Linear mixed-effects models examining the association between current SI and DMN activation during positive-versus-neutral contrasts of the Emotion N-back task without and with controlling for the effects of current MDD and ADHD.**](#_4d34og8) **11**

[**Table S7. Post-hoc analyses of the association between individual region activation of areas of the Salience Network, as defined by the destrieux atlas, and current suicide ideation (as compared to no suicide ideation). Each model included the covariates age, sex, race, family income band, and fMRI scanner platform. Displayed here are the regions with the three largest effect sizes for each task condition.**](#_nrh4c1h3zoqb) **12**

[**Table S8. Post-hoc analyses of the association between individual region activation of areas of the Default Mode Network, as defined by the destrieux atlas, and current suicide ideation (as compared to no suicide ideation). Each model included the covariates age, sex, race, family income band, and fMRI scanner platform. Displayed here are the regions with the six largest effect sizes.**](#_17dp8vu) **13**

[**Table S9. Sample descriptors based on group status (SI group defined as active SI only) and results from 2-tailed chi-squared and independent samples t-test analyses to determine group differences.**](#_26in1rg) **14**

[**Table S10. Linear mixed-effects models examining the association between SI (SI group defined as active SI only) and SN within-network RSFC without and with controlling for the effects of MDD, ADHD, and medications.**](#_lnxbz9) **15**

[**Table S11. Linear mixed-effects models examining the association between SI (SI group defined as active SI only) and DMN within-network RSFC without and with controlling for the effects of MDD, ADHD, and medications.**](#_35nkun2) **17**

[**Table S12. Linear mixed-effects models examining the association between SI (SI group defined as active SI only) and SN task activation to positive neutral and negative neutral contrasts without and with controlling for the effects of MDD, ADHD, and medications.**](#_1ksv4uv) **19**

[**Table S13. Linear mixed-effects models examining the association between SI (SI group defined as active SI only) and DMN task activation to positive neutral and negative neutral contrasts without and with controlling for the effects of MDD, ADHD, and medications.**](#_44sinio) **20**

[**Table S14. Sample descriptors based on group status (SI group defined as those who endorsed SI with no history of suicidal behavior) and results from 2-tailed chi-squared and independent samples t-test analyses to determine group differences.**](#_2jxsxqh) **21**

[**Table S15. Linear mixed-effects models examining the association between SI (SI group defined as those who endorsed SI with no history of suicidal behavior) and SN within-network RSFC without and with controlling for the effects of MDD, ADHD, and medications.**](#_mysqo1a1nnaj) **22**

[**Table S16. Linear mixed-effects models examining the association between SI (SI group defined as those who endorsed SI with no history of suicidal behavior) and DMN within-network RSFC without and with controlling for the effects of MDD, ADHD, and medications.**](#_3j2qqm3) **23**

[**Table S17. Linear mixed-effects models examining the association between SI (SI group defined as those who endorsed SI with no history of suicidal behavior) and SN task activation to positive neutral and negative neutral contrasts without and with controlling for the effects of MDD, ADHD, and medications.**](#_1y810tw) **24**

[**Table S18. Linear mixed-effects models examining the association between SI (SI group defined as those who endorsed SI with no history of suicidal behavior) and DMN task activation to positive neutral and negative neutral contrasts without and with controlling for the effects of MDD, ADHD, and medications.**](#_4i7ojhp) **26**

[**Table S19. Sample descriptors based on suicide ideation group status (as reported by either the parent or the child) and results from 2-tailed chi-squared and independent samples t-test analyses to determine group differences.**](#_2xcytpi) **28**

[**Table S20. Linear mixed-effects models examining the association between SI (as reported by either the parent or the child) and SN within-network RSFC without and with controlling for the effects of MDD, ADHD, and medications.**](#_japuxvu9x6g0) **29**

[**Table S21. Linear mixed-effects models examining the association between SI (as reported by either the parent or the child) and DMN within-network RSFC without and with controlling for the effects of MDD, ADHD, and medications.**](#_3whwml4) **30**

[**Table S22. Linear mixed-effects models examining the association between SI (as reported by either the parent or the child) and SN task activation to positive neutral and negative neutral contrasts without and with controlling for the effects of MDD, ADHD, and medications.**](#_2bn6wsx) **31**

[**Table S23. Linear mixed-effects models examining the association between SI (as reported by either the parent or the child) and DMN task activation to positive neutral and negative neutral contrasts without and with controlling for the effects of MDD, ADHD, and medications.**](#_qsh70q) **33**

[**Table S24. Sample descriptors based on suicide ideation group status (as reported by the parent only) and results from 2-tailed chi-squared and independent samples t-test analyses to determine group differences.**](#_3as4poj) **35**

[**Table S25. Linear mixed-effects models examining the association between SI (as reported by the parent only) and SN within-network RSFC without and with controlling for the effects of MDD, ADHD, and medications.**](#_mc175gfyve8g) **36**

[**Table S26. Linear mixed-effects models examining the association between SI (as reported by the parent only) and DMN within-network RSFC without and with controlling for the effects of MDD, ADHD, and medications.**](#_aflst9s09bnc) **37**

[**Table S27. Linear mixed-effects models examining the association between SI (as reported by the parent only) and SN task activation to positive neutral and negative neutral contrasts without and with controlling for the effects of MDD, ADHD, and medications.**](#_2p2csry) **38**

[**Table S28. Linear mixed-effects models examining the association between SI (as reported by the parent only) and DMN task activation to positive neutral and negative neutral contrasts without and with controlling for the effects of MDD, ADHD, and medications.**](#_147n2zr) **40**

[**Figure S1.**](#_jncpsa24a4r3) **42**

[**Figure S2.**](#_32hioqz) **43**

[Panel A. Post-hoc analyses of the association between pairwise resting-state connections of areas in the Salience Network, as defined by the Gordon atlas, and current suicide ideation (as compared to no suicide ideation). Each model included the covariates age, sex, race, family income band, and fMRI scanner platform. P-values were adjusted using the Hochberg procedure.](#_1hmsyys) 43

[Panel B. Individual regions that compose the Salience Network per the Gordon Atlas.](#_41mghml) 43

[**Figure S3.**](#_2grqrue) **44**

[Panel A. Post-hoc analyses of the association between pairwise resting-state connection of areas in the Default Mode Network, as defined by the Gordon atlas, and current suicide ideation (as compared to no suicide ideation). Each model included the covariates age, sex, race, family income band, and fMRI scanner platform. P-values were adjusted using the Hochberg procedure.](#_a7u632964vya) 44

[Panel B. Individual regions that compose the Default Mode Network per the Gordon Atlas. Regions depicted consist of those with the largest beta values in regression analyses where the pairwise connections were associated with suicide ideation.](#_vx1227) 44

# Supplemental Method.

**Measures**

*Medication.* Parent reports of their child’s prescription medications were gathered in the ABCD Parent Medications Survey Inventory and matched to classes within the Anatomical Therapeutic Chemical (ATC) classification using the National Library of Medicine RxClass API. Medications linked to the ATC code N06A were considered antidepressants, and medications linked to the ATC code N06B were considered stimulants.

# Table S1. Kiddie Schedule of Affective Disorders and Schizophrenia (KSADS) suicide ideation measures, and distribution of the study sample, across the following domains: passive, active but non-specific, specific method, active with intent, and active with a plan.

| **Construct** | **Question** | **N** |
| --- | --- | --- |
| Passive | Current: “In the past two weeks, how often have you wished you were dead or had thoughts that you would be better off dead?”  Past: “Was there ever a time in the past when you often wished you were dead or thought you would be better off dead?” | Current:  n = 85  Past:  n = 463 |
| Active nonspecific | Current: “In the past two weeks, how often did you think about wanting to kill yourself?”  Past: “Was there ever a time when you thought about wanting to kill yourself?” | Current:  n = 121  Past:  n = 272 |
| Active method | Current: “You mentioned in the past two weeks you thought about actually wanting to kill yourself. Have you thought about how you would do it (even if you had no intention of actually doing it)?”  Past: “You mentioned in the past you thought about actually wanting to kill yourself… Did you think about how you would do it (even if you had no intention of actually doing it)?” | Current:  n = 39  Past:  n = 64 |
| Active intent | Current: “At any point in the past two weeks did you have some intention on acting on these thoughts, even if you weren’t 100% sure you would do it?”  Past: “Back then, at any point did you have some intention of acting on these thoughts, even if you weren’t 100% sure you would do it?” | Current:  n = 20  Past:  n = 36 |
| Active plan | Current: “In the past two weeks, did you think through the details of how you would do it, for instance, decide on a specific method, place, or time?”  Past: “Did you think through the details of exactly how you would do it, for instance, decide on a specific place or time?” | Current:  n = 19  Past:  n = 24 |

#

# Table S2. Destrieux Atlas SN and DMN network regions

| Salience Network Regions |
| --- |
| anterior part of the cingulate gyrus (6) |
| anterior segment of the circular sulcus of insula (47) |
| superior segment of the circular sulcus of insula (49) |

| Default Mode Network Regions |
| --- |
| angular gyrus (25) |
| sulcus intermedius primus (55) |
| intraparietal sulcus (56) |
| superior temporal sulcus (73) |
| superior frontal sulcus (54) |
| posterior-dorsal part of the cingulate gyrus (9) |
| subparietal sulcus (71) |
| precuneus (30) |
| suborbital sulcus (70) |
| frontal superior gyrus (16) |
| straight gyrus (31) |
| middle frontal gyrus (15) |

# Table S3. Linear Mixed-Effects Models of the association between salience network within-network RSFC and SI without and with controlling for the effects of current Major Depressive Disorder and Attention-Deficit Hyperactivity Disorder.

Salience Network Resting-State Functional Connectivity

Model 1 Model 2

Main Effects *B* (se b) *B* (se b)

Age (in months)ª -0.01 (0.01) -0.01 (0.01)

Sex (REF = Female) 0.00 (0.02) 0.00 (0.02)

Race

White 0.11** (0.04) 0.11** (0.04)

Black 0.02 (0.04) 0.02 (0.04)

Asian 0.07 (0.05) 0.07 (0.05)

Native American -0.02 (0.06) -0.02 (0.06)

Hispanic/Latinx -0.02 (0.03) -0.02 (0.03)

Other Race 0.11* (0.06) 0.11* (0.06)

Family Income 0.03* (0.01) 0.03* (0.01)

Antidepressant Medication - - -0.11 (0.08)

Stimulant Medication - - -0.10* (0.05)

MDD (REF = No MDD History)

Past - -  *-*0.04 (0.09)

Current - - -0.15 (0.14)

ADHD (REF = No ADHD History)

Past - - 0.02 (0.04)

Current - - 0.06 (0.04)

Suicide ideation (REF = No SI History)

Past 0.01 (0.05) 0.02 (0.05)

Current -0.15 (0.08) -0.13 (0.08)

Primary Covariates *F^2^:* 0.000182742, Secondary Covariates *F^2^:* 0.000928992

*Note:* **p* < .05; ***p* < .01; *** p <.001; ª Pubertal status was also tested in addition to and in place of age. Results remained unchanged. SI = suicide ideation, SN= Salience Network, MDD= Major Depressive Disorder, ADHD= Attention-Deficit Hyperactivity Disorder, REF = Reference group for categorical variables.

# Table S4. Linear mixed-effects models examining the association between current SI and SN activation during positive-versus-neutral contrasts of the Emotion N-back task without and with controlling for the effects of current MDD and ADHD.

SN Positive V Neutral Task Activation

Model 1 Model 2

Main Effects *B* (se b) *B* (se b)

Age (in months)ª 0.00 (0.01) -0.00 (0.01)

Sex (REF = Female) 0.04 (0.02) 0.04 (0.02)

Race

White 0.00 (0.04) 0.00 (0.04)

Black -0.03 (0.04) -0.03 (0.04)

Asian 0.05 (0.05) 0.06 (0.05)

Native American -0.03 (0.06) -0.03 (0.06)

Hispanic/Latinx 0.04 (0.03) 0.04 (0.03)

Other Race 0.02 (0.06) 0.02 (0.06)

Family Income -0.01 (0.01) -0.01 (0.01)

Antidepressant Medication - - 0.06 (0.08)

Stimulant Medication - - 0.03 (0.05)

MDD (REF = No MDD History)

Past - - 0.03 (0.09)

Current - - -0.01 (0.14)

ADHD (REF = No ADHD History)

Past - - 0.05 (0.04)

Current - - -0.02 (0.04)

Suicide ideation (REF = No SI History)

Past -0.05 (0.05) -0.06 (0.05)

Current 0.06 (0.08) 0.06 (0.08)

Primary Covariates *F^2^:* 0.0001740065, Secondary Covariates *F^2^:* 0.0002944014

*Note:* **p* < .05; ***p* < .01; *** p <.001; ª Pubertal status was also tested in addition to and in place of age. Results remained unchanged. SI = suicide ideation, SN= Salience Network, MDD= Major Depressive Disorder, ADHD= Attention-Deficit Hyperactivity Disorder, REF = Reference group for categorical variables.

# Table S5. Linear mixed-effects models examining the association between current SI and SN activation during negative-versus-neutral contrasts of the Emotion N-back task without and with controlling for the effects of current MDD and ADHD.

SN Negative V Neutral Task Activation

Model 1 Model 2

Main Effects *B* (se b) *B* (se b)

Age (in months)ª -0.01 (0.01) -0.01 (0.01)

Sex (REF = Female) 0.04 (0.02) 0.04 (0.02)

Race

White -0.05 (0.04) -0.05 (0.04)

Black -0.03 (0.04) -0.03 (0.04)

Asian -0.02 (0.05) -0.02 (0.05)

Native American -0.15* (0.06) -0.15* (0.06)

Hispanic/Latinx -0.03 (0.03) -0.03 (0.03)

Other Race 0.12* (0.06) 0.12* (0.06)

Family Income 0.01 (0.01) 0.01 (0.01)

Antidepressant Medication - - 0.08 (0.08)

Stimulant Medication - - -0.05 (0.50)

MDD (REF = No MDD History)

Past - -  *-*0.01 (0.09)

Current - - 0.04 (0.14)

ADHD (REF = No ADHD History)

Past - - 0.05 (0.04)

Current - - 0.02 (0.04)

Suicide ideation (REF = No SI History)

Past -0.08 (0.05) -0.09 (0.05)

Current -0.03 (0.08) -0.04 (0.08)

Primary Covariates *F^2^:* 0.001633853, Secondary Covariates *F^2^:* 0.001729974

*Note:* **p* < .05; ***p* < .01; *** p <.001; ª Pubertal status was also tested in addition to and in place of age. Results remained unchanged. SI = suicide ideation, SN= Salience Network, MDD= Major Depressive Disorder, ADHD= Attention-Deficit Hyperactivity Disorder, REF = Reference group for categorical variables.

# Table S6. Linear mixed-effects models examining the association between current SI and DMN activation during positive-versus-neutral contrasts of the Emotion N-back task without and with controlling for the effects of current MDD and ADHD.

DMN Positive V Neutral Task Activation

Model 1 Model 2

Main Effects *B* (se b) *B* (se b)

Age (in months)ª 0.01 (0.01) 0.01 (0.01)

Sex (REF = Female) 0.00 (0.02) -0.00 (0.02)

Race

White 0.02 (0.04) 0.02 (0.04)

Black 0.03 (0.04) 0.03 (0.04)

Asian 0.08 (0.05) 0.09 (0.05)

Native American -0.04 (0.06) -0.03 (0.06)

Hispanic/Latinx 0.00 (0.03) 0.00 (0.03)

Other Race 0.07 (0.06) 0.07 (0.06)

Family Income -0.02 (0.01) -0.02 (0.01)

Antidepressant Medication - - -0.01 (0.08)

Stimulant Medication - - 0.06 (0.05)

MDD (REF = No MDD History)

Past - - 0.06 (0.09)

Current - - 0.12 (0.14)

ADHD (REF = No ADHD History)

Past - - 0.02 (0.04)

Current - - -0.01 (0.04)

Suicide ideation (REF = No SI History)

Past -0.07 (0.05) -0.07 (0.05)

Current -0.11 (0.08) -0.13 (0.08)

Primary Covariates *F^2^:* 0.0004841406, Secondary Covariates *F^2^:* 0.0007640024

*Note:* **p* < .05; ***p* < .01; *** p <.001; ª Pubertal status was also tested in addition to and in place of age. Results remained unchanged. SI = suicide ideation, DMN= Default Mode Network, MDD= Major Depressive Disorder, ADHD= Attention-Deficit Hyperactivity Disorder, REF = Reference group for categorical variables.

# Table S7. Post-hoc analyses of the association between individual region activation of areas of the Salience Network, as defined by the destrieux atlas, and current suicide ideation (as compared to no suicide ideation). Each model included the covariates age, sex, race, family income band, and fMRI scanner platform. Displayed here are the regions with the three largest effect sizes for each task condition.

| Contrast | Region of the Salience Network | *B* | SE | Adjusted P-value |
| --- | --- | --- | --- | --- |
| Positive versus Neutral contrasts | anterior segment of the circular sulcus of the insula | 0.08 | 0.08 | 0.96 |
|  | anterior part of the cingulate gyrus and sulcus | 0.06 | 0.08 | 0.96 |
|  | superior segment of the circular sulcus of the insula | 0.02 | 0.08 | 0.96 |
| Negative versus Neutral contrasts | anterior segment of the circular sulcus of the insula | -0.03 | 0.08 | 0.96 |
|  | anterior part of the cingulate gyrus and sulcus | -0.04 | 0.08 | 0.96 |
|  | superior segment of the circular sulcus of the insula | -0.00 | 0.08 | 0.96 |

# Table S8. Post-hoc analyses of the association between individual region activation of areas of the Default Mode Network, as defined by the destrieux atlas, and current suicide ideation (as compared to no suicide ideation). Each model included the covariates age, sex, race, family income band, and fMRI scanner platform. Displayed here are the regions with the six largest effect sizes.

| Contrast | Region of the Default Mode Network | *B* | SE | Adjusted P-value |
| --- | --- | --- | --- | --- |
| Negative versus Neutral contrasts | subparietal sulcus | -0.25 | 0.08 | 0.03 |
|  | superior temporal sulcus | -0.27 | 0.08 | 0.01 |
|  | posterior dorsal part of the cingulate gyrus | -0.26 | 0.08 | 0.02 |
|  | suborbital sulcus | -0.17 | 0.08 | 0.54 |
|  | precuneus | -0.16 | 0.08 | 0.94 |
| Positive versus Neutral contrasts | posterior dorsal part of the cingulate gyrus | -0.19 | 0.08 | 0.30 |

#

# Table S9. Sample descriptors based on group status (SI group defined as active SI only) and results from 2-tailed chi-squared and independent samples t-test analyses to determine group differences.

|  | Never SI | | Past SI | | Current SI | | p-value |
| --- | --- | --- | --- | --- | --- | --- | --- |
|  | n = 7898 | | n = 229 | | n = 121 | |  |
| Age in Months, M (SD) | 119.22 | -7.52 | 119.53 | -7.69 | 117.85 | -7.03 | 0.11 |
| Total Income, M (SD)^a^ | 7.38 | -2.33 | 7.1 | -2.33 | 6.95 | -2.58 | 0.03 |
| Sex, N (%) |  |  |  |  |  |  |  |
| Male | 3987 | 50.48% | 133 | 58.08% | 69 | 57.02% | 0.03 |
| Female | 3911 | 49.52% | 96 | 41.92% | 52 | 42.98% | 0.03 |
| Race/ Ethnicity, N (%)^b^ |  |  |  |  |  |  |  |
| Asian | 544 | 6.89% | 20 | 8.73% | 9 | 7.44% | 0.54 |
| Black | 1387 | 17.56% | 54 | 23.58% | 24 | 19.83% | 0.05 |
| Latinx/Hispanic | 1528 | 19.35% | 30 | 13.10% | 32 | 26.45% | 0.01 |
| Native American | 246 | 3.11% | 12 | 5.24% | 8 | 6.61% | 0.02 |
| White | 6223 | 78.79% | 174 | 75.98% | 87 | 71.90% | 0.11 |
| Other Race | 467 | 5.91% | 8 | 3.49% | 11 | 9.09% | 0.10 |
| On Antidepressants, N(%) | 123 | 1.56% | 23 | 10.04% | 5 | 4.13% | <.001 |
| On Stimulants, N(%) | 558 | 7.07% | 36 | 15.72% | 13 | 10.74% | <.001 |
| MDD Past, N(%) | 104 | 1.32% | 20 | 8.73% | 7 | 5.79% | <.001 |
| MDD Current, N(%) | 39 | 0.49% | 6 | 2.62% | 6 | 4.96% | <.001 |
| ADHD Past, N(%) | 688 | 8.71% | 33 | 14.41% | 17 | 14.05% | <.001 |
| ADHD Current, N(%) | 770 | 9.75% | 43 | 18.78% | 24 | 19.83% | <.001 |

*Note:* ^a^ Total income bands: 1= Less than $5,000; 2=$5,000 - $11,999; 3=$12,000 - $15,999; 4=$16,000 - $24,999; 5=$25,000 - $34,999; 6=$35,000 - $49,999; 7=$50,000 - $74,999; 8= $75,000 - $99,999; 9=$100,000 - $199,999; 10=$200,000 and greater. ^b^ We allowed for participant overlap in the coding of racial or ethnic identity, where multiracial/multiethnic participants are represented in all relevant categories. *Abbreviations:* MDD= Major Depressive Disorder, ADHD= Attention-Deficit Hyperactivity Disorder.

# Table S10. Linear mixed-effects models examining the association between SI (SI group defined as active SI only) and SN within-network RSFC without and with controlling for the effects of MDD, ADHD, and medications.

SN RSFC

|  | Model 1 |  | Model 2 |  |
| --- | --- | --- | --- | --- |
|  | B | (se b) | B | (se b) |
| Age (in months) | -0.01 | 0.01 | -0.01 | 0.01 |
| Family Income | 0.03* | 0.01 | 0.03* | 0.01 |
| Sex (REF = Male) | 0.00 | 0.02 | 0.00 | 0.02 |
| Race |  |  |  |  |
| Asian | 0.07 | 0.05 | 0.07 | 0.05 |
| Black | 0.02 | 0.04 | 0.02 | 0.04 |
| Hispanic/ latinx | -0.02 | 0.03 | -0.02 | 0.03 |
| Native American | -0.02 | 0.06 | -0.03 | 0.06 |
| White | 0.11** | 0.04 | 0.11** | 0.04 |
| Other Race | 0.12* | 0.06 | 0.11* | 0.06 |
| Antidepressant Medication | - | - | -0.11 | 0.08 |
| Stimulant Medication | - | - | -0.10* | 0.05 |
| MDD (REF = No MDD History) | |  |  |  |
| Past | - | - | -0.05 | 0.09 |
| Current | - | - | -0.17 | 0.14 |
| ADHD (REF = No ADHD History) | |  |  |  |
| Past | - | - | 0.02 | 0.04 |
| Current | - | - | 0.06 | 0.04 |
| SI (REF = No SI History) | |  |  |  |
| Past | 0.04 | 0.07 | 0.06 | 0.07 |
| Current | -0.07 | 0.09 | -0.07 | 0.09 |

*Note:* **p* < .05; ***p* < .01; *** p <.001; SI = suicide ideation, RSFC = resting state functional connectivity, SN= Salience Network, MDD= Major Depressive Disorder, ADHD= Attention-Deficit Hyperactivity Disorder, REF = Reference group for categorical variables.

# Table S11. Linear mixed-effects models examining the association between SI (SI group defined as active SI only) and DMN within-network RSFC without and with controlling for the effects of MDD, ADHD, and medications.

DMN RSFC

|  | Model 1 |  | Model 2 |  |
| --- | --- | --- | --- | --- |
|  | B | (se b) | B | (se b) |
| Age (in months) | 0.05*** | 0.01 | 0.05*** | 0.01 |
| Family Income | 0.02 | 0.01 | 0.02 | 0.01 |
| Sex (REF = Male) | -0.25*** | 0.02 | -0.25*** | 0.02 |
| Race |  |  |  |  |
| Asian | -0.01 | 0.04 | -0.02 | 0.04 |
| Black | -0.20*** | 0.04 | -0.20*** | 0.04 |
| Hispanic/ latinx | -0.02 | 0.03 | -0.02 | 0.03 |
| Native American | 0.06 | 0.06 | 0.06 | 0.06 |
| White | 0.04 | 0.04 | 0.04 | 0.04 |
| Other Race | -0.01 | 0.05 | -0.01 | 0.05 |
| Antidepressant Medication | - | - | -0.07 | 0.08 |
| Stimulant Medication | - | - | 0.07 | 0.05 |
| MDD (REF = No MDD History) | |  |  |  |
| Past | - | - | -0.06 | 0.09 |
| Current | - | - | -0.49*** | 0.14 |
| ADHD (REF = No ADHD History) | |  |  |  |
| Past | - | - | -0.04 | 0.04 |
| Current | - | - | -0.10** | 0.04 |
| SI (REF = No SI History) | |  |  |  |
| Past | -0.01 | 0.06 | 0.02 | 0.07 |
| Current | -0.25** | 0.09 | -0.22* | 0.09 |

*Note:* **p* < .05; ***p* < .01; *** p <.001; SI = suicide ideation, RSFC = resting state functional connectivity, DMN= Default Mode Network, MDD= Major Depressive Disorder, ADHD= Attention-Deficit Hyperactivity Disorder, REF = Reference group for categorical variables.

# Table S12. Linear mixed-effects models examining the association between SI (SI group defined as active SI only) and SN task activation to positive neutral and negative neutral contrasts without and with controlling for the effects of MDD, ADHD, and medications.

SN PVN SN NVN

|  | Model | 1 | Model | 2 | Model | 1 | Model | 2 |
| --- | --- | --- | --- | --- | --- | --- | --- | --- |
|  | B | (se b) | B | (se b) | B | (se b) | B | (se b) |
| Age (in months) | 0.00 | 0.01 | 0.00 | 0.01 | -0.01 | 0.01 | -0.01 | 0.01 |
| Family Income | -0.01 | 0.01 | -0.01 | 0.01 | 0.01 | 0.01 | 0.01 | 0.01 |
| Sex (REF = Male) | 0.04 | 0.02 | 0.04 | 0.02 | 0.04 | 0.02 | 0.04 | 0.02 |
| Race |  |  |  |  |  |  |  |  |
| Asian | 0.05 | 0.05 | 0.05 | 0.05 | -0.02 | 0.05 | -0.02 | 0.05 |
| Black | -0.03 | 0.04 | -0.03 | 0.04 | -0.03 | 0.04 | -0.03 | 0.04 |
| Hispanic/ latinx | 0.04 | 0.03 | 0.04 | 0.03 | -0.03 | 0.03 | -0.03 | 0.03 |
| Native American | -0.03 | 0.06 | -0.03 | 0.06 | -0.15* | 0.06 | -0.15* | 0.06 |
| White | 0.00 | 0.04 | 0.00 | 0.04 | -0.05 | 0.04 | -0.05 | 0.04 |
| Other Race | 0.02 | 0.06 | 0.02 | 0.06 | 0.12* | 0.06 | 0.12* | 0.06 |
| Antidepressant Medication- | | - | 0.05 | 0.08 | - | - | 0.07 | 0.08 |
| Stimulant Medication | - | - | 0.03 | 0.05 | - | - | -0.05 | 0.05 |
| MDD (REF = No MDD History) | | |  |  |  |  |  |  |
| Past | - | - | 0.02 | 0.09 | - | - | -0.01 | 0.09 |
| Current | - | - | 0.00 | 0.14 | - | - | 0.04 | 0.14 |
| ADHD (REF = No ADHD History) | | |  |  |  |  |  |  |
| Past | - | - | 0.05 | 0.04 | - | - | 0.05 | 0.04 |
| Current | - | - | -0.02 | 0.04 | - | - | 0.02 | 0.04 |
| SI (REF = No SI History) | |  |  |  |  |  |  |  |
| Past | 0.02 | 0.07 | 0.01 | 0.07 | -0.10 | 0.07 | -0.11 | 0.07 |
| Current | -0.02 | 0.09 | -0.02 | 0.09 | -0.07 | 0.09 | -0.08 | 0.09 |

*Note:* **p* < .05; ***p* < .01; *** p <.001; SI = suicide ideation, RSFC = resting state functional connectivity, SN= Salience Network, MDD= Major Depressive Disorder, ADHD= Attention-Deficit Hyperactivity Disorder, REF = Reference group for categorical variables.

# Table S13. Linear mixed-effects models examining the association between SI (SI group defined as active SI only) and DMN task activation to positive neutral and negative neutral contrasts without and with controlling for the effects of MDD, ADHD, and medications.

DMN PVN DMN NVN

|  | Model | 1 | Model | 2 | Model | 1 | Model | 2 |
| --- | --- | --- | --- | --- | --- | --- | --- | --- |
|  | B | (se b) | B | (se b) | B | (se b) | B | (se b) |
| Age (in months) | 0.01 | 0.01 | 0.01 | 0.01 | 0.00 | 0.01 | 0.00 | 0.01 |
| Family Income | -0.02 | 0.01 | -0.02 | 0.01 | -0.01 | 0.01 | -0.01 | 0.01 |
| Sex (REF = Male) | 0.00 | 0.02 | 0.00 | 0.02 | 0.00 | 0.02 | -0.01 | 0.02 |
| Race |  |  |  |  |  |  |  |  |
| Asian | 0.07 | 0.05 | 0.08 | 0.05 | 0.01 | 0.05 | 0.01 | 0.05 |
| Black | 0.03 | 0.04 | 0.03 | 0.04 | 0.07 | 0.04 | 0.07 | 0.04 |
| Hispanic/ latinx | 0.00 | 0.03 | 0.00 | 0.03 | -0.05 | 0.03 | -0.05 | 0.03 |
| Native American | -0.04 | 0.06 | -0.04 | 0.06 | -0.08 | 0.06 | -0.08 | 0.06 |
| White | 0.02 | 0.04 | 0.02 | 0.04 | 0.05 | 0.04 | 0.04 | 0.04 |
| Other Race | 0.07 | 0.06 | 0.07 | 0.06 | 0.14* | 0.06 | 0.14* | 0.06 |
| Antidepressant Medication - | | - | -0.03 | 0.08 | - | - | 0.05 | 0.08 |
| Stimulant Medication | - | - | 0.06 | 0.05 | - | - | 0.03 | 0.05 |
| MDD (REF = No MDD History) | |  |  |  |  |  |  |  |
| Past | - | - | 0.04 | 0.09 | - | - | 0.04 | 0.09 |
| Current | - | - | 0.10 | 0.14 | - | - | 0.06 | 0.14 |
| ADHD (REF = No ADHD History) | |  |  |  |  |  |  |  |
| Past | - | - | 0.02 | 0.04 | - | - | 0.06 | 0.04 |
| Current | - | - | -0.02 | 0.04 | - | - | 0.05 | 0.04 |
| SI (REF = No SI History) | |  |  |  |  |  |  |  |
| Past | 0.03 | 0.07 | 0.03 | 0.07 | -0.04 | 0.07 | -0.06 | 0.07 |
| Current | -0.17 | 0.09 | -0.17 | 0.09 | -0.19* | 0.09 | -0.21* | 0.09 |

*Note:* **p* < .05; ***p* < .01; *** p <.001; SI = suicide ideation, RSFC = resting state functional connectivity, DMN= Default Mode Network, MDD= Major Depressive Disorder, ADHD= Attention-Deficit Hyperactivity Disorder, REF = Reference group for categorical variables.

# Table S14. Sample descriptors based on group status (SI group defined as those who endorsed SI with no history of suicidal behavior) and results from 2-tailed chi-squared and independent samples t-test analyses to determine group differences.

|  | Never SI | | Past SI | | Current SI | | p-value |
| --- | --- | --- | --- | --- | --- | --- | --- |
|  | n = 7579 | | n = 442 | | n = 135 | |  |
| Age in Months, M (SD) | 119.22 | -7.52 | 119.54 | -7.64 | 117.24 | -6.73 | 0.01 |
| Total Income, M (SD)^a^ | 7.38 | -2.33 | 7.47 | -2.17 | 7.05 | -2.51 | 0.19 |
| Sex, N (%) |  |  |  |  |  |  |  |
| Male | 3821 | 50.42% | 235 | 53.17% | 82 | 60.74% | 0.03 |
| Female | 3758 | 49.58% | 207 | 46.83% | 53 | 39.26% | 0.03 |
| Race, N (%)^b^ |  |  |  |  |  |  |  |
| Asian | 522 | 6.89% | 36 | 8.14% | 10 | 7.41% | 0.59 |
| Black | 1329 | 17.54% | 88 | 19.91% | 24 | 17.78% | 0.44 |
| Hispanic | 1473 | 19.44% | 60 | 13.57% | 31 | 22.96% | 0.01 |
| Native American | 233 | 3.07% | 24 | 5.43% | 9 | 6.67% | <.01 |
| White | 5973 | 78.81% | 345 | 78.05% | 102 | 75.56% | 0.62 |
| Other race | 451 | 5.95% | 21 | 4.75% | 9 | 6.67% | 0.54 |
| On Antidepressants, N (%) | 108 | 1.42% | 27 | 6.11% | 4 | 2.96% | <.001 |
| On Stimulants, N (%) | 528 | 6.97% | 47 | 10.63% | 15 | 11.11% | <.001 |
| MDD Past, N (%) | 87 | 1.15% | 29 | 6.56% | 4 | 2.96% | <.001 |
| MDD Current, N (%) | 31 | 0.41% | 3 | 0.68% | 12 | 8.89% | <.001 |
| ADHD Past, N (%) | 639 | 8.43% | 67 | 15.16% | 20 | 14.81% | <.001 |
| ADHD Current, N (%) | 733 | 9.67% | 62 | 14.03% | 21 | 15.56% | <.001 |

*Note:* ^a^ Total income bands: 1= Less than $5,000; 2=$5,000 - $11,999; 3=$12,000 - $15,999; 4=$16,000 - $24,999; 5=$25,000 - $34,999; 6=$35,000 - $49,999; 7=$50,000 - $74,999; 8= $75,000 - $99,999; 9=$100,000 - $199,999; 10=$200,000 and greater. ^b^ We allowed for participant overlap in the coding of racial or ethnic identity, where multiracial/multiethnic participants are represented in all relevant categories. *Abbreviations:* MDD= Major Depressive Disorder, ADHD= Attention-Deficit Hyperactivity Disorder.

# Table S15. Linear mixed-effects models examining the association between SI (SI group defined as those who endorsed SI with no history of suicidal behavior) and SN within-network RSFC without and with controlling for the effects of MDD, ADHD, and medications.

SN RSFC

|  | Model 1 |  | Model 2 |  |
| --- | --- | --- | --- | --- |
|  | B | (se b) | B | (se b) |
| Age (in months) | -0.01 | 0.01 | -0.01 | 0.01 |
| Family Income | 0.02 | 0.01 | 0.02 | 0.01 |
| Sex (REF = Male) | 0.01 | 0.02 | 0.01 | 0.02 |
| Race |  |  |  |  |
| Asian | 0.07 | 0.05 | 0.07 | 0.05 |
| Black | 0.01 | 0.04 | 0.02 | 0.04 |
| Hispanic/ latinx | -0.02 | 0.03 | -0.02 | 0.03 |
| Native American | -0.03 | 0.06 | -0.03 | 0.06 |
| White | 0.11** | 0.04 | 0.11** | 0.04 |
| Other Race | 0.12* | 0.06 | 0.12* | 0.06 |
| Antidepressant Medication | - | - | -0.14 | 0.09 |
| Stimulant Medication | - | - | -0.09 | 0.05 |
| MDD (REF = No MDD History) | |  |  |  |
| Past | - | - | -0.05 | 0.09 |
| Current | - | - | -0.18 | 0.15 |
| ADHD (REF = No ADHD History) | |  |  |  |
| Past | - | - | 0.02 | 0.04 |
| Current | - | - | 0.07 | 0.04 |
| SI (REF = No SI History) | |  |  |  |
| Past | 0.04 | 0.05 | 0.04 | 0.05 |
| Current | -0.12 | 0.09 | -0.10 | 0.09 |

*Note:* **p* < .05; ***p* < .01; *** p <.001; SI = suicide ideation, RSFC = resting state functional connectivity, SN= Salience Network, MDD= Major Depressive Disorder, ADHD= Attention-Deficit Hyperactivity Disorder, REF = Reference group for categorical variables.

# Table S16. Linear mixed-effects models examining the association between SI (SI group defined as those who endorsed SI with no history of suicidal behavior) and DMN within-network RSFC without and with controlling for the effects of MDD, ADHD, and medications.

DMN RSFC

|  | Model 1 |  | Model 2 |  |
| --- | --- | --- | --- | --- |
|  | B | (se b) | B | (se b) |
| Age (in months) | 0.05*** | 0.01 | 0.05*** | 0.01 |
| Family Income | 0.01 | 0.01 | 0.01 | 0.01 |
| Sex (REF = Male) | -0.25*** | 0.02 | -0.24*** | 0.02 |
| Race |  |  |  |  |
| Asian | -0.02 | 0.04 | -0.02 | 0.04 |
| Black | -0.21*** | 0.04 | -0.20*** | 0.04 |
| Hispanic/ latinx | -0.02 | 0.03 | -0.02 | 0.03 |
| Native American | 0.06 | 0.06 | 0.06 | 0.06 |
| White | 0.03 | 0.04 | 0.03 | 0.04 |
| Other Race | -0.02 | 0.05 | -0.02 | 0.05 |
| Antidepressant Medication | - | - | -0.07 | 0.08 |
| Stimulant Medication | - | - | 0.06 | 0.05 |
| MDD (REF = No MDD History) | |  |  |  |
| Past | - | - | -0.05 | 0.09 |
| Current | - | - | -0.47** | 0.14 |
| ADHD (REF = No ADHD History) | |  |  |  |
| Past | - | - | -0.05 | 0.04 |
| Current | - | - | -0.11** | 0.04 |
| SI (REF = No SI History) | |  |  |  |
| Past | 0.05 | 0.05 | 0.06 | 0.05 |
| Current | -0.24** | 0.08 | -0.20* | 0.08 |

*Note:* **p* < .05; ***p* < .01; *** p <.001; SI = suicide ideation, RSFC = resting state functional connectivity, DMN= Default Mode Network, MDD= Major Depressive Disorder, ADHD= Attention-Deficit Hyperactivity Disorder, REF = Reference group for categorical variables.

# Table S17. Linear mixed-effects models examining the association between SI (SI group defined as those who endorsed SI with no history of suicidal behavior) and SN task activation to positive neutral and negative neutral contrasts without and with controlling for the effects of MDD, ADHD, and medications.

SN PVN SN NVN

|  | Model | 1 | Model | 2 | Model | 1 | Model | 2 |
| --- | --- | --- | --- | --- | --- | --- | --- | --- |
|  | B | (se b) | B | (se b) | B | (se b) | B | (se b) |
| Age (in months) | 0.00 | 0.01 | 0.00 | 0.01 | -0.01 | 0.01 | -0.01 | 0.01 |
| Family Income | -0.01 | 0.01 | -0.01 | 0.01 | 0.01 | 0.01 | 0.01 | 0.01 |
| Sex (REF = Male) | 0.04 | 0.02 | 0.04 | 0.02 | 0.04 | 0.02 | 0.04 | 0.02 |
| Race |  |  |  |  |  |  |  |  |
| Asian | 0.05 | 0.05 | 0.05 | 0.05 | -0.02 | 0.05 | -0.02 | 0.05 |
| Black | -0.03 | 0.04 | -0.03 | 0.04 | -0.02 | 0.04 | -0.02 | 0.04 |
| Hispanic/ latinx | 0.03 | 0.03 | 0.03 | 0.03 | -0.03 | 0.03 | -0.03 | 0.03 |
| Native American | -0.03 | 0.06 | -0.03 | 0.06 | -0.15* | 0.06 | -0.15* | 0.06 |
| White | 0.00 | 0.04 | 0.00 | 0.04 | -0.04 | 0.04 | -0.04 | 0.04 |
| Other Race | 0.03 | 0.06 | 0.03 | 0.06 | 0.13* | 0.06 | 0.13* | 0.06 |
| Antidepressant Medication - | | - | 0.07 | 0.09 | - | - | 0.12 | 0.09 |
| Stimulant Medication | - | - | 0.01 | 0.05 | - | - | -0.06 | 0.05 |
| MDD (REF = No MDD History) | | |  |  |  |  |  |  |
| Past | - | - | 0.03 | 0.09 | - | - | 0.00 | 0.09 |
| Current | - | - | -0.03 | 0.15 | - | - | 0.00 | 0.15 |
| ADHD (REF = No ADHD History) | | |  |  |  |  |  |  |
| Past | - | - | 0.06 | 0.04 | - | - | 0.06 | 0.04 |
| Current | - | - | 0.00 | 0.04 | - | - | 0.03 | 0.04 |
| SI (REF = No SI History) | |  |  |  |  |  |  |  |
| Past | -0.02 | 0.05 | -0.03 | 0.05 | -0.06 | 0.05 | -0.07 | 0.05 |
| Current | 0.00 | 0.09 | 0.00 | 0.09 | -0.14 | 0.09 | -0.15 | 0.09 |

*Note:* **p* < .05; ***p* < .01; *** p <.001; SI = suicide ideation, RSFC = resting state functional connectivity, SN= Salience Network, MDD= Major Depressive Disorder, ADHD= Attention-Deficit Hyperactivity Disorder, REF = Reference group for categorical variables.

# Table S18. Linear mixed-effects models examining the association between SI (SI group defined as those who endorsed SI with no history of suicidal behavior) and DMN task activation to positive neutral and negative neutral contrasts without and with controlling for the effects of MDD, ADHD, and medications.

DMN PVN DMN NVN

|  | Model | 1 | Model | 2 | Model | 1 | Model | 2 |
| --- | --- | --- | --- | --- | --- | --- | --- | --- |
|  | B | (se b) | B | (se b) | B | (se b) | B | (se b) |
| Age (in months) | 0.01 | 0.01 | 0.01 | 0.01 | 0.00 | 0.01 | 0.00 | 0.01 |
| Family Income | -0.02 | 0.01 | -0.02 | 0.01 | -0.01 | 0.01 | -0.01 | 0.01 |
| Sex (REF = Male) | 0.00 | 0.02 | 0.00 | 0.02 | -0.01 | 0.02 | -0.01 | 0.02 |
| Race |  |  |  |  |  |  |  |  |
| Asian | 0.07 | 0.05 | 0.07 | 0.05 | 0.00 | 0.05 | 0.00 | 0.05 |
| Black | 0.03 | 0.04 | 0.03 | 0.04 | 0.08 | 0.04 | 0.07 | 0.04 |
| Hispanic/ latinx | 0.00 | 0.03 | 0.00 | 0.03 | -0.05 | 0.03 | -0.04 | 0.03 |
| Native American | -0.03 | 0.06 | -0.03 | 0.06 | -0.08 | 0.06 | -0.08 | 0.06 |
| White | 0.03 | 0.04 | 0.03 | 0.04 | 0.05 | 0.04 | 0.05 | 0.04 |
| Other Race | 0.06 | 0.06 | 0.06 | 0.06 | 0.14* | 0.06 | 0.14* | 0.06 |
| Antidepressant Medication - | | - | -0.02 | 0.09 | - | - | 0.03 | 0.09 |
| Stimulant Medication | - | - | 0.05 | 0.05 | - | - | 0.04 | 0.05 |
| MDD (REF = No MDD History) | |  |  |  |  |  |  |  |
| Past | - | - | 0.06 | 0.09 | - | - | 0.07 | 0.09 |
| Current | - | - | 0.03 | 0.15 | - | - | 0.00 | 0.15 |
| ADHD (REF = No ADHD History) | | |  |  |  |  |  |  |
| Past | - | - | 0.03 | 0.04 | - | - | 0.07 | 0.04 |
| Current | - | - | -0.01 | 0.04 | - | - | 0.05 | 0.04 |
| SI (REF = No SI History) | |  |  |  |  |  |  |  |
| Past | -0.07 | 0.05 | -0.07 | 0.05 | -0.03 | 0.05 | -0.04 | 0.05 |
| Current | -0.10 | 0.09 | -0.11 | 0.09 | -0.19* | 0.09 | -0.20* | 0.09 |

*Note:* **p* < .05; ***p* < .01; *** p <.001; SI = suicide ideation, RSFC = resting state functional connectivity, DMN= Default Mode Network, MDD= Major Depressive Disorder, ADHD= Attention-Deficit Hyperactivity Disorder, REF = Reference group for categorical variables.

# Table S19. Sample descriptors based on suicide ideation group status (as reported by either the parent or the child) and results from 2-tailed chi-squared and independent samples t-test analyses to determine group differences.

|  | Never SI | | Past SI | | Current SI | | p- value |
| --- | --- | --- | --- | --- | --- | --- | --- |
|  | n = 7155 | | n = 842 | | n = 251 | |  |
| Age in Months, M (SD) | 119.23 | -7.52 | 119.31 | -7.58 | 118.21 | -7.12 | 0.1 |
| Total Income, M (SD)^a^ | 7.4 | -2.33 | 7.33 | -2.26 | 6.65 | -2.6 | < 0.01 |
| Sex, N (%) |  |  |  |  |  |  |  |
| Male | 3555 | 49.69% | 478 | 56.77% | 156 | 62.15% | < 0.01 |
| Female | 3600 | 50.31% | 364 | 43.23% | 95 | 37.85% | < 0.01 |
| Race, N (%)^b^ |  |  |  |  |  |  |  |
| Asian | 490 | 6.85% | 65 | 7.72% | 18 | 7.17% | 0.64 |
| Black | 1249 | 17.46% | 160 | 19.00% | 56 | 22.31% | 0.09 |
| Hispanic | 1388 | 19.40% | 144 | 17.10% | 58 | 23.11% | 0.08 |
| Native American | 218 | 3.05% | 35 | 4.16% | 13 | 5.18% | 0.05 |
| White | 5620 | 78.55% | 678 | 80.52% | 186 | 74.10% | 0.09 |
| Other race | 418 | 5.84% | 52 | 6.18% | 16 | 6.37% | 0.88 |
| On Antidepressants, N (%) | 81 | 1.13% | 54 | 6.41% | 16 | 6.37% | < 0.01 |
| On Stimulants, N (%) | 455 | 6.36% | 114 | 13.54% | 38 | 15.14% | < 0.01 |
| MDD Past, N (%) | 176 | 2.46% | 106 | 12.59% | 33 | 13.15% | < 0.01 |
| MDD Current, N (%) | 35 | 0.49% | 17 | 2.02% | 19 | 7.57% | < 0.01 |
| ADHD Past, N (%) | 583 | 8.15% | 113 | 13.42% | 42 | 16.73% | < 0.01 |
| ADHD Current, N (%) | 615 | 8.60% | 160 | 19.00% | 62 | 24.70% | < 0.01 |

*Note:* ^a^ Total income bands: 1= Less than $5,000; 2=$5,000 - $11,999; 3=$12,000 - $15,999; 4=$16,000 - $24,999; 5=$25,000 - $34,999; 6=$35,000 - $49,999; 7=$50,000 - $74,999; 8= $75,000 - $99,999; 9=$100,000 - $199,999; 10=$200,000 and greater. ^b^ We allowed for participant overlap in the coding of racial or ethnic identity, where multiracial/multiethnic participants are represented in all relevant categories. *Abbreviations:* MDD= Major Depressive Disorder, ADHD= Attention-Deficit Hyperactivity Disorder.

# Table S20. Linear mixed-effects models examining the association between SI (as reported by either the parent or the child) and SN within-network RSFC without and with controlling for the effects of MDD, ADHD, and medications.

SN RFSC

|  | Model 1 |  | Model 2 |  |
| --- | --- | --- | --- | --- |
|  | B | (se b) | B | (se b) |
| Age (in months) | -0.01 | 0.01 | -0.01 | 0.01 |
| Family Income | 0.03* | 0.01 | 0.03* | 0.01 |
| Sex (REF = Male) | 0.00 | 0.02 | 0.00 | 0.02 |
| Race |  |  |  |  |
| Asian | 0.07 | 0.05 | 0.07 | 0.05 |
| Black | 0.02 | 0.04 | 0.02 | 0.04 |
| Hispanic/ latinx | -0.02 | 0.03 | -0.02 | 0.03 |
| Native American | -0.02 | 0.06 | -0.02 | 0.06 |
| White | 0.11** | 0.04 | 0.11** | 0.04 |
| Other Race | 0.11* | 0.06 | 0.11* | 0.06 |
| Antidepressant Medication | - | - | -0.11 | 0.08 |
| Stimulant Medication | - | - | -0.10* | 0.05 |
| MDD (REF = No MDD History) | |  |  |  |
| Past | - | - | 0.01 | 0.06 |
| Current | - | - | -0.07 | 0.12 |
| ADHD (REF = No ADHD History) | |  |  |  |
| Past | - | - | 0.02 | 0.04 |
| Current | - | - | 0.06 | 0.04 |
| SI (REF = No SI History) | |  |  |  |
| Past | 0.02 | 0.04 | 0.02 | 0.04 |
| Current | -0.08 | 0.06 | -0.08 | 0.07 |

*Note:* **p* < .05; ***p* < .01; *** p <.001; SI = suicide ideation, RSFC = resting state functional connectivity, SN= Salience Network, MDD= Major Depressive Disorder, ADHD= Attention-Deficit Hyperactivity Disorder, REF = Reference group for categorical variables.

# Table S21. Linear mixed-effects models examining the association between SI (as reported by either the parent or the child) and DMN within-network RSFC without and with controlling for the effects of MDD, ADHD, and medications.

DMN RSFC

|  | Model 1 |  | Model 2 |  |
| --- | --- | --- | --- | --- |
|  | B | (se b) | B | (se b) |
| Age (in months) | 0.05*** | 0.01 | 0.05*** | 0.01 |
| Family Income | 0.02 | 0.01 | 0.01 | 0.01 |
| Sex (REF = Male) | -0.25*** | 0.02 | -0.25*** | 0.02 |
| Race |  |  |  |  |
| Asian | -0.01 | 0.04 | -0.02 | 0.04 |
| Black | -0.20*** | 0.04 | -0.20*** | 0.04 |
| Hispanic/ latinx | -0.02 | 0.03 | -0.02 | 0.03 |
| Native American | 0.06 | 0.06 | 0.06 | 0.06 |
| White | 0.04 | 0.04 | 0.05 | 0.04 |
| Other Race | -0.01 | 0.05 | -0.01 | 0.05 |
| Antidepressant Medication | - | - | -0.06 | 0.08 |
| Stimulant Medication | - | - | 0.06 | 0.05 |
| MDD (REF = No MDD History) | |  |  |  |
| Past | - | - | -0.10 | 0.06 |
| Current | - | - | -0.28* | 0.12 |
| ADHD (REF = No ADHD History) | |  |  |  |
| Past | - | - | -0.04 | 0.04 |
| Current | - | - | -0.10* | 0.04 |
| SI (REF = No SI History) | |  |  |  |
| Past | 0.01 | 0.04 | 0.04 | 0.04 |
| Current | -0.21*** | 0.06 | -0.17** | 0.06 |

*Note:* **p* < .05; ***p* < .01; *** p <.001; SI = suicide ideation, RSFC = resting state functional connectivity, DMN= Default Mode Network, MDD= Major Depressive Disorder, ADHD= Attention-Deficit Hyperactivity Disorder, REF = Reference group for categorical variables.

# Table S22. Linear mixed-effects models examining the association between SI (as reported by either the parent or the child) and SN task activation to positive neutral and negative neutral contrasts without and with controlling for the effects of MDD, ADHD, and medications.

SN PVN SN NVN

|  | Model | 1 | Model | 2 | Model | 1 | Model | 2 |
| --- | --- | --- | --- | --- | --- | --- | --- | --- |
|  | B | (se b) | B | (se b) | B | (se b) | B | (se b) |
| Age (in months) | 0.00 | 0.01 | 0.00 | 0.01 | -0.01 | 0.01 | -0.01 | 0.01 |
| Family Income | -0.01 | 0.01 | -0.01 | 0.01 | 0.01 | 0.01 | 0.01 | 0.01 |
| Sex (REF = Male) | 0.04 | 0.02 | 0.04 | 0.02 | 0.04 | 0.02 | 0.04 | 0.02 |
| Race |  |  |  |  |  |  |  |  |
| Asian | 0.05 | 0.05 | 0.05 | 0.05 | -0.02 | 0.05 | -0.02 | 0.05 |
| Black | -0.03 | 0.04 | -0.03 | 0.04 | -0.03 | 0.04 | -0.03 | 0.04 |
| Hispanic/ latinx | 0.04 | 0.03 | 0.04 | 0.03 | -0.03 | 0.03 | -0.03 | 0.03 |
| Native American | -0.03 | 0.06 | -0.03 | 0.06 | -0.15* | 0.06 | -0.15* | 0.06 |
| White | 0.00 | 0.04 | 0.00 | 0.04 | -0.05 | 0.04 | -0.05 | 0.04 |
| Other Race | 0.02 | 0.06 | 0.02 | 0.06 | 0.12* | 0.06 | 0.12* | 0.06 |
| Antidepressant Medication - | | - | 0.04 | 0.08 | - | - | 0.07 | 0.08 |
| Stimulant Medication | - | - | 0.03 | 0.05 | - | - | -0.05 | 0.05 |
| MDD (REF = No MDD History) | | |  |  |  |  |  |  |
| Past | - | - | 0.03 | 0.06 | - | - | -0.02 | 0.06 |
| Current | - | - | -0.02 | 0.12 | - | - | 0.08 | 0.12 |
| ADHD (REF = No ADHD History) | | |  |  |  |  |  |  |
| Past | - | - | 0.05 | 0.04 | - | - | 0.05 | 0.04 |
| Current | - | - | -0.02 | 0.04 | - | - | 0.02 | 0.04 |
| SI (REF = No SI History) | |  |  |  |  |  |  |  |
| Past | 0.01 | 0.04 | 0.00 | 0.04 | -0.05 | 0.04 | -0.05 | 0.04 |
| Current | 0.03 | 0.06 | 0.02 | 0.07 | -0.02 | 0.06 | -0.03 | 0.07 |

*Note:* **p* < .05; ***p* < .01; *** p <.001; SI = suicide ideation, RSFC = resting state functional connectivity, SN= Salience Network, MDD= Major Depressive Disorder, ADHD= Attention-Deficit Hyperactivity Disorder, REF = Reference group for categorical variables

# Table S23. Linear mixed-effects models examining the association between SI (as reported by either the parent or the child) and DMN task activation to positive neutral and negative neutral contrasts without and with controlling for the effects of MDD, ADHD, and medications.

DMN PVN DMN NVN

|  | Model | 1 | Model | 2 | Model | 1 | Model | 2 |
| --- | --- | --- | --- | --- | --- | --- | --- | --- |
|  | B | (se b) | B | (se b) | B | (se b) | B | (se b) |
| Age (in months) | 0.01 | 0.01 | 0.01 | 0.01 | 0.00 | 0.01 | 0.00 | 0.01 |
| Family Income | -0.02 | 0.01 | -0.02 | 0.01 | -0.01 | 0.01 | -0.01 | 0.01 |
| Sex (REF = Male) | 0.00 | 0.02 | 0.00 | 0.02 | 0.00 | 0.02 | -0.01 | 0.02 |
| Race |  |  |  |  |  |  |  |  |
| Asian | 0.08 | 0.05 | 0.08 | 0.05 | 0.01 | 0.05 | 0.01 | 0.05 |
| Black | 0.03 | 0.04 | 0.03 | 0.04 | 0.07 | 0.04 | 0.07 | 0.04 |
| Hispanic/ latinx | 0.00 | 0.03 | 0.00 | 0.03 | -0.05 | 0.03 | -0.05 | 0.03 |
| Native American | -0.04 | 0.06 | -0.04 | 0.06 | -0.08 | 0.06 | -0.09 | 0.06 |
| White | 0.03 | 0.04 | 0.02 | 0.04 | 0.05 | 0.04 | 0.04 | 0.04 |
| Other Race | 0.07 | 0.06 | 0.07 | 0.06 | 0.14* | 0.06 | 0.14* | 0.06 |
| Antidepressant Medication - | | - | -0.03 | 0.08 | - | - | 0.05 | 0.08 |
| Stimulant Medication | - | - | 0.06 | 0.05 | - | - | 0.03 | 0.05 |
| MDD (REF = No MDD History) | |  |  |  |  |  |  |  |
| Past | - | - | 0.05 | 0.06 | - | - | 0.00 | 0.06 |
| Current | - | - | 0.12 | 0.12 | - | - | 0.02 | 0.12 |
| ADHD (REF = No ADHD History) | | |  |  |  |  |  |  |
| Past | - | - | 0.02 | 0.04 | - | - | 0.06 | 0.04 |
| Current | - | - | -0.02 | 0.04 | - | - | 0.05 | 0.04 |
| SI (REF = No SI History) | |  |  |  |  |  |  |  |
| Past | 0.00 | 0.04 | 0.00 | 0.04 | 0.03 | 0.04 | 0.01 | 0.04 |
| Current | -0.10 | 0.06 | -0.11 | 0.07 | -0.11 | 0.06 | -0.13* | 0.07 |

*Note:* **p* < .05; ***p* < .01; *** p <.001; SI = suicide ideation, RSFC = resting state functional connectivity, DMN= Default Mode Network, MDD= Major Depressive Disorder, ADHD= Attention-Deficit Hyperactivity Disorder, REF = Reference group for categorical variables.

# Table S24. Sample descriptors based on suicide ideation group status (as reported by the parent only) and results from 2-tailed chi-squared and independent samples t-test analyses to determine group differences.

|  | Never SI | | Past SI | | Current SI | | p-value |
| --- | --- | --- | --- | --- | --- | --- | --- |
|  | n = 7630 | | n = 510 | | n = 108 | |  |
| Age in Months, M (SD) | 119.19 | -7.51 | 119.54 | -7.57 | 118.69 | -7.55 | 0.47 |
| Total Income, M (SD)^a^ | 7.38 | -2.33 | 7.33 | -2.28 | 6.43 | -2.61 | <0.01 |
| Sex, N (%) |  |  |  |  |  |  |  |
| Male | 3802 | 49.83% | 315 | 61.76% | 72 | 66.67% | <0.01 |
| Female | 3828 | 50.17% | 195 | 38.24% | 36 | 33.33% | <0.01 |
| Race, N (%)^b^ |  |  |  |  |  |  |  |
| Asian | 526 | 6.89% | 40 | 7.84% | 7 | 6.48% | 0.7 |
| Black | 1345 | 17.63% | 92 | 18.04% | 28 | 25.93% | 0.08 |
| Hispanic | 1476 | 19.34% | 95 | 18.63% | 19 | 17.59% | 0.84 |
| Native American | 240 | 3.15% | 19 | 3.73% | 7 | 6.48% | 0.12 |
| White | 5983 | 78.41% | 422 | 82.75% | 79 | 73.15% | 0.03 |
| Other race | 438 | 5.74% | 41 | 8.04% | 7 | 6.48% | 0.10 |
| On Antidepressants, N (%) | 96 | 1.26% | 43 | 8.43% | 12 | 11.11% | <0.01 |
| On Stimulants, N (%) | 501 | 6.57% | 82 | 16.08% | 24 | 22.22% | <0.01 |
| MDD Past, N (%) | 111 | 1.45% | 68 | 13.33% | 12 | 11.11% | <0.01 |
| MDD Current, N (%) | 10 | 0.13% | 6 | 1.18% | 12 | 11.11% | <0.01 |
| ADHD Past, N (%) | 642 | 8.41% | 75 | 14.71% | 21 | 19.44% | <0.01 |
| ADHD Current, N (%) | 667 | 8.74% | 130 | 25.49% | 40 | 37.04% | <0.01 |

*Note:* ^a^ Total income bands: 1= Less than $5,000; 2=$5,000 - $11,999; 3=$12,000 - $15,999; 4=$16,000 - $24,999; 5=$25,000 - $34,999; 6=$35,000 - $49,999; 7=$50,000 - $74,999; 8= $75,000 - $99,999; 9=$100,000 - $199,999; 10=$200,000 and greater. ^b^ We allowed for participant overlap in the coding of racial or ethnic identity, where multiracial/multiethnic participants are represented in all relevant categories. *Abbreviations:* MDD= Major Depressive Disorder, ADHD= Attention-Deficit Hyperactivity Disorder.

# Table S25. Linear mixed-effects models examining the association between SI (as reported by the parent only) and SN within-network RSFC without and with controlling for the effects of MDD, ADHD, and medications.

SN RFSC

|  | Model 1 |  | Model 2 |  |
| --- | --- | --- | --- | --- |
|  | B | (se b) | B | (se b) |
| Age (in months) | -0.01 | 0.01 | -0.01 | 0.01 |
| Family Income | 0.03* | 0.01 | 0.03* | 0.01 |
| Sex (REF = Male) | 0.00 | 0.02 | 0.00 | 0.02 |
| Race |  |  |  |  |
| Asian | 0.07 | 0.05 | 0.07 | 0.05 |
| Black | 0.02 | 0.04 | 0.02 | 0.04 |
| Hispanic/ latinx | -0.02 | 0.03 | -0.02 | 0.03 |
| Native American | -0.02 | 0.06 | -0.03 | 0.06 |
| White | 0.11** | 0.04 | 0.11** | 0.04 |
| Other Race | 0.11* | 0.06 | 0.11* | 0.06 |
| Antidepressant Medication | - | - | -0.12 | 0.08 |
| Stimulant Medication | - | - | -0.10* | 0.05 |
| MDD (REF = No MDD History) | |  |  |  |
| Past | - | - | 0.03 | 0.08 |
| Current | - | - | 0.13 | 0.19 |
| ADHD (REF = No ADHD History) | |  |  |  |
| Past | - | - | 0.02 | 0.04 |
| Current | - | - | 0.05 | 0.04 |
| SI (REF = No SI History) | |  |  |  |
| Past | 0.04 | 0.05 | 0.04 | 0.05 |
| Current | 0.08 | 0.10 | 0.07 | 0.10 |

*Note:* **p* < .05; ***p* < .01; *** p <.001; SI = suicide ideation, RSFC = resting state functional connectivity, SN= Salience Network, MDD= Major Depressive Disorder, ADHD= Attention-Deficit Hyperactivity Disorder, REF = Reference group for categorical variables.

# Table S26. Linear mixed-effects models examining the association between SI (as reported by the parent only) and DMN within-network RSFC without and with controlling for the effects of MDD, ADHD, and medications.

DMN RSFC

|  | Model 1 |  | Model 2 |  |
| --- | --- | --- | --- | --- |
|  | B | (se b) | B | (se b) |
| Age (in months) | 0.05*** | 0.01 | 0.05*** | 0.01 |
| Family Income | 0.02 | 0.01 | 0.02 | 0.01 |
| Sex (REF = Male) | -0.25*** | 0.02 | -0.25*** | 0.02 |
| Race |  |  |  |  |
| Asian | -0.01 | 0.04 | -0.02 | 0.04 |
| Black | -0.20*** | 0.04 | -0.20*** | 0.04 |
| Hispanic/ latinx | -0.02 | 0.03 | -0.02 | 0.03 |
| Native American | 0.06 | 0.06 | 0.06 | 0.06 |
| White | 0.04 | 0.04 | 0.05 | 0.04 |
| Other Race | -0.01 | 0.05 | -0.01 | 0.05 |
| Antidepressant Medication | - | - | -0.07 | 0.08 |
| Stimulant Medication | - | - | 0.07 | 0.05 |
| MDD (REF = No MDD History) | |  |  |  |
| Past | - | - | -0.12 | 0.07 |
| Current | - | - | 0.23 | 0.19 |
| ADHD (REF = No ADHD History) | |  |  |  |
| Past | - | - | -0.05 | 0.04 |
| Current | - | - | -0.11** | 0.04 |
| SI (REF = No SI History) | |  |  |  |
| Past | 0.00 | 0.04 | 0.03 | 0.05 |
| Current | -0.09 | 0.09 | -0.08 | 0.10 |

*Note:* **p* < .05; ***p* < .01; *** p <.001; SI = suicide ideation, RSFC = resting state functional connectivity, DMN= Default Mode Network, MDD= Major Depressive Disorder, ADHD= Attention-Deficit Hyperactivity Disorder, REF = Reference group for categorical variables.

# Table S27. Linear mixed-effects models examining the association between SI (as reported by the parent only) and SN task activation to positive neutral and negative neutral contrasts without and with controlling for the effects of MDD, ADHD, and medications.

SN PVN SN NVN

|  | Model | 1 | Model | 2 | Model | 1 | Model | 2 |
| --- | --- | --- | --- | --- | --- | --- | --- | --- |
|  | B | (se b) | B | (se b) | B | (se b) | B | (se b) |
| Age (in months) | 0.00 | 0.01 | 0.00 | 0.01 | -0.01 | 0.01 | -0.01 | 0.01 |
| Family Income | -0.01 | 0.01 | -0.01 | 0.01 | 0.01 | 0.01 | 0.01 | 0.01 |
| Sex (REF = Male) | 0.04 | 0.02 | 0.04 | 0.02 | 0.04 | 0.02 | 0.04 | 0.02 |
| Race |  |  |  |  |  |  |  |  |
| Asian | 0.05 | 0.05 | 0.05 | 0.05 | -0.02 | 0.05 | -0.02 | 0.05 |
| Black | -0.03 | 0.04 | -0.03 | 0.04 | -0.03 | 0.04 | -0.03 | 0.04 |
| Hispanic/ latinx | 0.04 | 0.03 | 0.04 | 0.03 | -0.03 | 0.03 | -0.03 | 0.03 |
| Native American | -0.03 | 0.06 | -0.03 | 0.06 | -0.15* | 0.06 | -0.15* | 0.06 |
| White | -0.01 | 0.04 | -0.01 | 0.04 | -0.05 | 0.04 | -0.05 | 0.04 |
| Other Race | 0.02 | 0.06 | 0.02 | 0.06 | 0.12* | 0.06 | 0.12* | 0.06 |
| Antidepressant Medication - | | - | 0.03 | 0.08 | - | - | 0.06 | 0.08 |
| Stimulant Medication | - | - | 0.03 | 0.05 | - | - | -0.05 | 0.05 |
| MDD (REF = No MDD History) | | |  |  |  |  |  |  |
| Past | - | - | 0.04 | 0.08 | - | - | -0.02 | 0.08 |
| Current | - | - | 0.07 | 0.19 | - | - | 0.17 | 0.19 |
| ADHD (REF = No ADHD History) | | |  |  |  |  |  |  |
| Past | - | - | 0.04 | 0.04 | - | - | 0.04 | 0.04 |
| Current | - | - | -0.02 | 0.04 | - | - | 0.01 | 0.04 |
| SI (REF = No SI History) | |  |  |  |  |  |  |  |
| Past | 0.08 | 0.05 | 0.08 | 0.05 | -0.01 | 0.05 | -0.02 | 0.05 |
| Current | -0.05 | 0.10 | -0.07 | 0.10 | 0.02 | 0.10 | -0.01 | 0.10 |

*Note:* **p* < .05; ***p* < .01; *** p <.001; SI = suicide ideation, RSFC = resting state functional connectivity, SN= Salience Network, MDD= Major Depressive Disorder, ADHD= Attention-Deficit Hyperactivity Disorder, REF = Reference group for categorical variables.

# Table S28. Linear mixed-effects models examining the association between SI (as reported by the parent only) and DMN task activation to positive neutral and negative neutral contrasts without and with controlling for the effects of MDD, ADHD, and medications.

DMN PVN DMN NVN

|  | Model | 1 | Model | 2 | Model | 1 | Model | 2 |
| --- | --- | --- | --- | --- | --- | --- | --- | --- |
|  | B | (se b) | B | (se b) | B | (se b) | B | (se b) |
| Age (in months) | 0.01 | 0.01 | 0.01 | 0.01 | 0.00 | 0.01 | 0.00 | 0.01 |
| Family Income | -0.02 | 0.01 | -0.02 | 0.01 | -0.01 | 0.01 | -0.01 | 0.01 |
| Sex (REF = Male) | 0.00 | 0.02 | 0.00 | 0.02 | -0.01 | 0.02 | -0.01 | 0.02 |
| Race |  |  |  |  |  |  |  |  |
| Asian | 0.07 | 0.05 | 0.07 | 0.05 | 0.00 | 0.05 | 0.01 | 0.05 |
| Black | 0.03 | 0.04 | 0.03 | 0.04 | 0.07 | 0.04 | 0.07 | 0.04 |
| Hispanic/ latinx | 0.00 | 0.03 | 0.00 | 0.03 | -0.05 | 0.03 | -0.05 | 0.03 |
| Native American | -0.04 | 0.06 | -0.04 | 0.06 | -0.09 | 0.06 | -0.09 | 0.06 |
| White | 0.02 | 0.04 | 0.02 | 0.04 | 0.04 | 0.04 | 0.04 | 0.04 |
| Other Race | 0.06 | 0.06 | 0.06 | 0.06 | 0.14* | 0.06 | 0.14* | 0.06 |
| Antidepressant Medication - | | - | -0.04 | 0.08 | - | - | 0.03 | 0.08 |
| Stimulant Medication | - | - | 0.06 | 0.05 | - | - | 0.03 | 0.05 |
| MDD (REF = No MDD History) | |  |  |  |  |  |  |  |
| Past | - | - | 0.02 | 0.08 | - | - | -0.04 | 0.08 |
| Current | - | - | 0.19 | 0.19 | - | - | 0.05 | 0.19 |
| ADHD (REF = No ADHD History) | | |  |  |  |  |  |  |
| Past | - | - | 0.01 | 0.04 | - | - | 0.05 | 0.04 |
| Current | - | - | -0.02 | 0.04 | - | - | 0.04 | 0.04 |
| SI (REF = No SI History) | |  |  |  |  |  |  |  |
| Past | 0.07 | 0.05 | 0.06 | 0.05 | 0.07 | 0.05 | 0.06 | 0.05 |
| Current | -0.06 | 0.10 | -0.08 | 0.10 | 0.09 | 0.10 | 0.07 | 0.10 |

*Note:* **p* < .05; ***p* < .01; *** p <.001; SI = suicide ideation, RSFC = resting state functional connectivity, DMN= Default Mode Network, MDD= Major Depressive Disorder, ADHD= Attention-Deficit Hyperactivity Disorder, REF = Reference group for categorical variables.

# Figure S1.

Consort diagram describing final study sample and missing data a







##



# Figure S2.

A.

| Dependent Variable  (pairwise connections) | *B* | SE | Adjusted  P-value |
| --- | --- | --- | --- |
| 29 Left and 183 Right | 0.24 | 0.12 | 0.26 |
| 183 Right and 247 Right | 0.05 | 0.12 | 0.88 |
| 29 Left and 83 Left | -0.08 | 0.12 | 0.88 |
| 83 Left and 183 Right | -0.02 | 0.12 | 0.88 |
| 83 Left and 247 Right | -0.04 | 0.11 | 0.88 |
| 29 Left and 247 Right | -0.09 | 0.12 | 0.88 |

B.

##
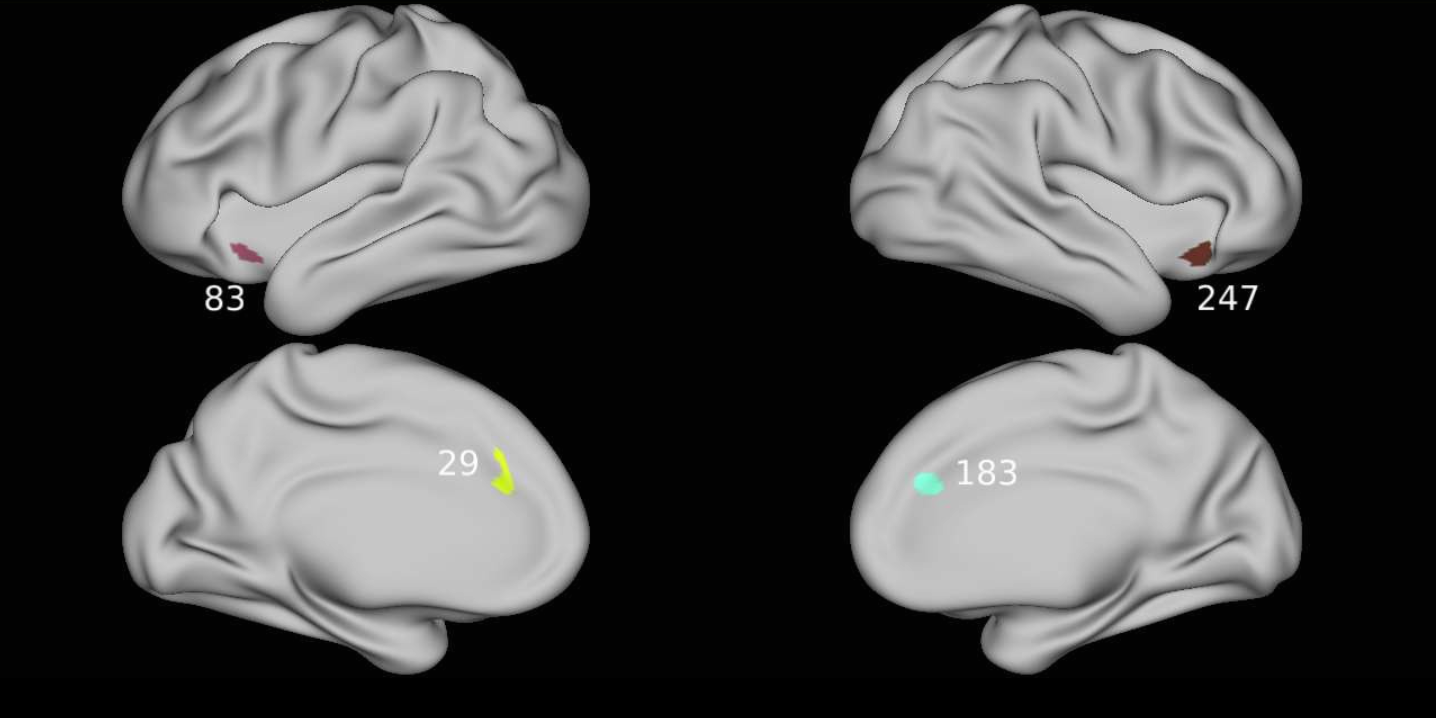


## Panel A. Post-hoc analyses of the association between pairwise resting-state connections of areas in the Salience Network, as defined by the Gordon atlas, and current suicide ideation (as compared to no suicide ideation). Each model included the covariates age, sex, race, family income band, and fMRI scanner platform. P-values were adjusted using the Hochberg procedure.

## Panel B. Individual regions that compose the Salience Network per the Gordon Atlas.

# Figure S3.

A.

| Dependent Variable  (pairwise connections) | *B* | SE | Adjusted  P-value |
| --- | --- | --- | --- |
| 126 Left and 324 Right | -0.46 | 0.12 | 0.09 |
| 126 Left and 145 Right | -0.44 | 0.12 | 0.18 |
| 44 Left and 290 Right | -0.45 | 0.11 | 0.09 |
| 126 Left and 257 Right | 0.41 | 0.12 | 0.51 |
| 94 Left and 331 Right | -0.40 | 0.12 | 0.64 |
| 165 Left and 257 Right | 0.39 | 0.12 | 0.76 |
| 44 Left and 331 Right | -0.40 | 0.12 | 0.76 |

B.

**
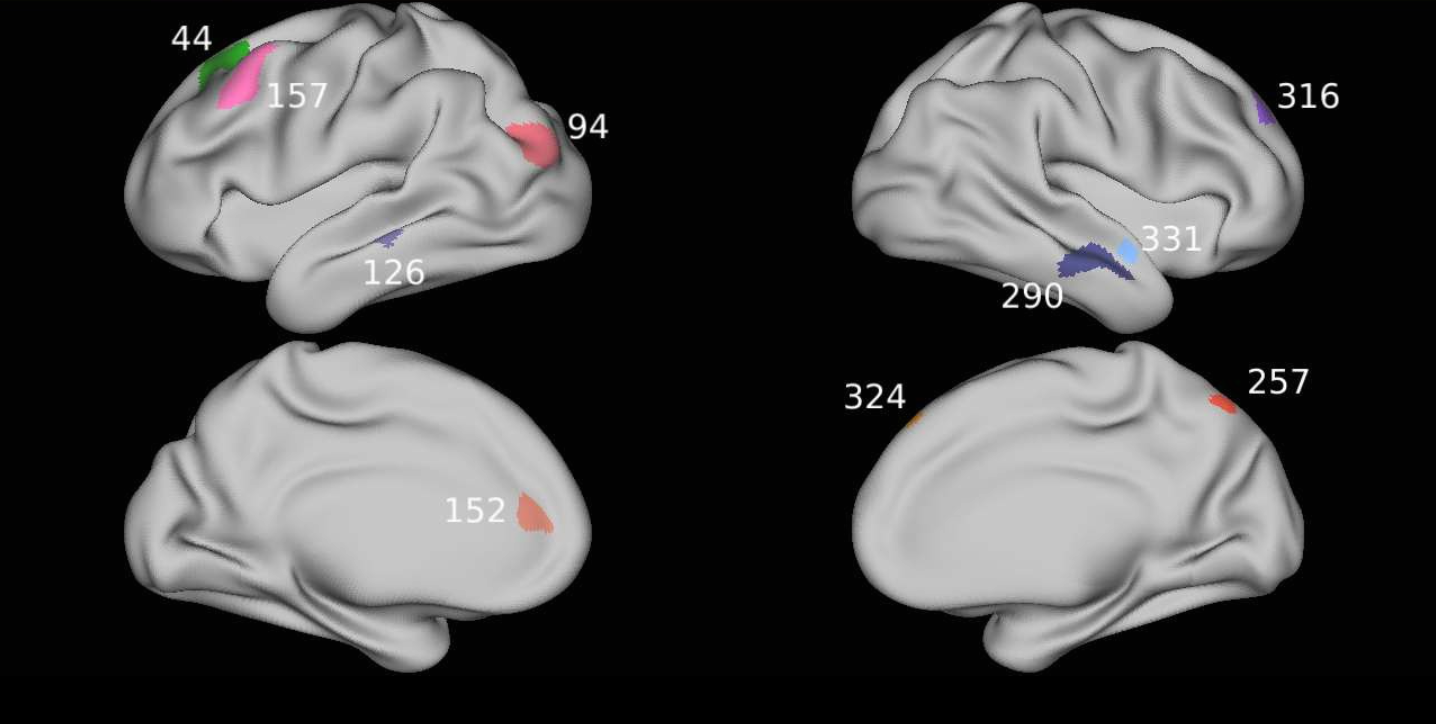
**

## Panel A. Post-hoc analyses of the association between pairwise resting-state connection of areas in the Default Mode Network, as defined by the Gordon atlas, and current suicide ideation (as compared to no suicide ideation). Each model included the covariates age, sex, race, family income band, and fMRI scanner platform. P-values were adjusted using the Hochberg procedure.

## Panel B. Individual regions that compose the Default Mode Network per the Gordon Atlas. Regions depicted consist of those with the largest beta values in regression analyses where the pairwise connections were associated with suicide ideation.
